# SVD-clustering, a general image-analyzing method explained and demonstrated on model and Raman micro-spectroscopic maps

**DOI:** 10.1038/s41598-020-61206-9

**Published:** 2020-03-06

**Authors:** B. Szalontai, M. Debreczeny, K. Fintor, Cs. Bagyinka

**Affiliations:** 1 Institute of Biophysics, Biological Research Centre of the Hungarian Academy of Sciences, Szeged, Hungary; 2 BOKU-VIBT Imaging Centre, University of Natural Resources and Life Sciences, Vienna, Austria; 3 ‘Vulcano’ Petrology and Geochemistry Research Group, Department of Mineralogy, Geochemistry and Petrology, Faculty of Science and Informatics, University of Szeged, Szeged, Hungary

**Keywords:** Imaging, Computational biophysics, Image processing

## Abstract

An image analyzing method (SVD-clustering) is presented. Amplitude vectors of SVD factorization (V_1_…V_i_) were introduced into the imaging of the distribution of the corresponding U_i_ basis-spectra. Since each V_i_ vector contains each point of the map, plotting them along the X, Y, Z dimensions of the map reconstructs the spatial distribution of the corresponding U_i_ basis-spectrum. This gives valuable information about the first, second, etc. higher-order deviations present in the map. We extended SVD with a clustering method, using the significant V_i_ vectors from the V^T^ matrix as coordinates of image points in a n_e_-dimensional space (n_e_ is the effective rank of the data matrix). This way every image point had a corresponding coordinate in the n_e_-dimensional space and formed a point set. Clustering was applied to this point set. SVD-clustering is universal; it is applicable to any measurement where data are recorded as a function of an external parameter (time, space, temperature, concentration, species, etc.). Consequently, our method is not restricted to spectral imaging, it can find application in many different 2D and 3D image analyses. Using SVD-clustering, we have shown on models the theoretical possibilities and limitations of the method, especially in the context of creating, meaning/interpreting of cluster spectra. Then for real-world samples, two examples are presented, where we were able to reveal minute alterations in the samples (changing cation ratios in minerals, differently structured cellulose domains in plant root) with spatial resolution.

## Introduction

The image is a spatial representation of an object by a physical, chemical or biological property. The ‘property’ practically can be anything which has a value (e.g. intensity of light, the concentration of a compound, etc.) or distribution (e.g. visible, fluorescence or Raman spectra, etc.) at each point of the object. The best-known example is the simple photography where light intensity and color is assigned to each spatial point. Images became very popular when new instruments, producing both 2D and 3D images, were developed and the use of them turned into common. Numerous imaging techniques are currently in use in order to visualize objects. Different microscopic techniques were industrialized, medical imaging (CT, fMRI, and PET) are more and more frequent.

Several of the techniques use labeled molecules (PET, laser scanning flurescent microscopy, etc.), thus the information obtained might be biased by the different – unwanted - interactions of the labeled compound. Others (confocal Raman or infrared imaging, fMRI, CT, X-ray, etc.) are methods to map objects using non-invasive techniques (e.g. inelastic scattering, infrared spectroscopy, magnetic field, etc.)^1–3^.

Using microscopes, the new detecting systems (e.g. CCD cameras) and the confocal technology together with a high precision scanning stage allow sensitive and high spatial resolution imaging of samples (down to the diffraction limit of the applied light) with high signal-to-noise ratios and thus visualize the intracellular components.

If the ‘property’ is just a value, evaluation of the image is quite straightforward because only one interpretation is possible. Evaluation and interpretation of an image become difficult if the ‘property’ attached to every spatial point has a ‘distribution’, e.g. the ‘property’ is a spectrum, time dependence, etc. Several methods have been developed to get information in visual form. The simplest case, if a special point or a value calculated from an interval of the distribution is used to reproduce the image this way reducing the distribution to a single value. For example, a single peak or a specific spectral region is selected from the spectrum and the intensity of the region/peak is attached to every spatial point thus producing an image.

Global methods try to use all the information buried in the measurement. These computational techniques attempt to extract information using the whole ‘distribution’ and reconstruct different images from different parts of the data^4^. Such algorithms were mostly introduced in analytical chemistry and include multivariate approaches like Orthogonal Projection Approach (OPA)^5^, SIMPLE-to-use Self-modeling Mixture Analysis (SIMPLISMA)^6^, Multivariate Curve Resolution – Alternating Least Squares (MCR-ALS)^7–11^, Positive Matrix Factorization (PMF)^12^, Non-Negative Matrix Factorization (NNMF)^13,14^, and Hyperspectral Image Analysis (HIA)^15,16^. Besides these techniques, well-known global data manipulation methods like SVD and PCA^4^ are frequently used to simplify the data sets and filter noise.

Some of these methods also have promised to de-convolute ‘real’ component spectra from the map. These are multivariate self-modeling methods like MCR-ALS^7–11^, SIMPLISMA^6^, OPA^5^ and many others. All are using an iteration procedure to de-convolute the large set of different spectra obtained at spatial points of the image. The result of the iteration is a concentration matrix and a component spectra matrix.

The ‘global’ methods can be divided into supervised, and unsupervised groups. Unsupervised methods, do not assume any *a priori* condition on the data. All manipulations performed on the data obey only to the rules of the applied mathematics. Supervised methods need users’ decisions in order to perform the task (e.g. determination of significant components etc.).

SVD analysis in Raman spectroscopy has already proven to be useful in revealing ‘hidden’ components in complex systems, e.g. in the case of lipid fatty acyl chain conformational changes, when it could show that under the phenomenological frequency shift of the v_sym_(CH_2_) band there are the opposing intensity changes of two-component bands^17^. In an even more complicated situation, when one single lipid double layer was embedded in between two large polyelectrolyte multilayers domain, the temperature-dependent phase transition of the lipid bilayer fatty acyl chain could be ‘surfaced’^18^.

In a more recent, biological application, Raman spectra were recorded on apoptotic cells, and SVD analysis was used to filter out the noise from the data. SVD analysis made possible to check the possible laser damage in the cells and was used to characterize them^19^. The spatial distribution of SVD basis spectra could be used to reveal component distribution in core/shell nanofibers^20^, and to determine compartments in live cells^21–23^. These publications have only made use of the noise-filtering capacity of the SVD, no attempt has been done to use the unsupervised information provided by the method for further analysis of the experimental data.

Clustering was already used in the evaluation of image maps^23^. In this case, the pairwise similarity of spectra was used as a point-group (similarity was usually calculated using the Pearson correlation). An earlier approach used PCA analysis^24,25^ and applied clustering on scores and determined cluster spectra.

Here, we present an SVD-based evaluation method (SVD-clustering), which takes into account the full information provided by the SVD analysis.

We use (i) the distributions of the different U_i_ basis-spectra, by plotting their amplitude vectors (V_i_ vectors) along the scanned area. This way not only the distribution of the average intensities but also the meaningful spectral deviations can be visualized at a much better signal-to-noise ratio. (ii) Based on the results of the unsupervised SVD analysis, a supervised method, a *cluster analysis*, is introduced having much higher confidence, since the selection of the clusters is based on the similarity of the distribution of low-noise V_i_ vectors (obtained without any pre-condition). Thus, the characteristic spectra of different sample regions can be reconstructed and studied independently.

We present the theoretical sensitivity of the new method on models and compare PCA- and SVD-clustering.

Finally, we show the extreme sensitivity of the SVD analysis to changes in chemical composition on a geological sample and the distribution and the variability of cellulose molecules - their conformational and chemical inhomogeneity - at different locations in a root section of the *Catharanthus roseus* plant.

## Materials and methods

### Model calculations

All calculations on images were performed using a home-built software written in Matlab. We used the R2018a version with no special toolboxes.

#### SVD map calculations

SVD factorize the D data matrix

$${\rm{D}}={\rm{U}}\ast {\rm{W}}\ast {{\rm{V}}}^{{\rm{T}}}$$

(For details about SVD analysis, see Supplementary Information 1).

Images were reconstructed from the rows of the **V**^**T**^ matrix (we call them as V vectors) by using the (x, y, z) coordinates, associated with every column of the **V**^**T**^ matrix. This way we got several maps (V_i_-maps), each corresponding to different rows of the **V**^**T**^ matrix (V_1_, V_2_, …. V_n_). The elements of **W** (W_ii_ and their relative value W_ii_/ΣW) determined the contribution of the corresponding V_i_-maps to the overall picture of the map. Since the W_ii_ elements are sorted out in decreasing order, the first map (V_1_-map) gives the highest contribution, usually between 50–99%, depending on the sample and on the noise of the measurement. Thus, the first map gives an ‘average’ picture of the sample, which is similar to a conventional map, generated by using spectrum integrals. Contributions of subsequent V_i_-maps are usually much smaller (~1–10%), as they represent the first, second, etc. deviations from the average. The weights are usually decreasing quite fast, even a complex biological sample had rarely more than 10 significant V_i_ vectors. The i-value of the last significant W_ii_ represents the effective rank (n_e_) of the given measurement. All subsequent V vectors could be considered as noise. To determine the effective rank is crucial in the evaluation process.

As an additional rule, if there was no observable structure on a V_i_-map, just homogeneous noise, this V_i_ vector was also neglected even if its W_ii_ value was relatively high.

The columns of **U** matrix (U_i_ vectors), representing the orthonormal spectra of the original measurement, were also used in the evaluation. Here U_1_ represented the average spectrum of the entire scanned area of the sample, and it is very similar to the spectrum obtained by conventional analysis. U_2_ represented the first, while U_3_-U_i_ represented the second and higher order deviations from the average.

#### Clustering the map points

As a new element in SVD data analysis, we used the values of the significant V_i_ vectors for clustering the map points. If we take V_i_ vectors as multidimensional coordinates, this representation puts every map point into an n_e_-dimensional space where clusters can be specified using a proper distance measure. Once the **V**^**T**^ matrix was calculated, any clustering method could be used. We have tried several clustering and distance methods/functions; their results were not differing considerably, their differences rather seemed to depend on the noise of the measurement and on the properties of the sample. We did not make a thorough study in order to find the best clustering method and distance function. Thus, selecting the best clustering method should depend on the actual data sets. We used *k-means* clustering and Euclidean distance throughout the paper.

Since clustering is not an exact method, rather an interactive multi-objective optimization that involves trial and failure, it is the user’s task to determine the exact conditions for clustering. It is, however, obvious that the maximal dimension (how many V_i_ vectors should be considered) should not exceed the significant number of V_i_-maps (i.e. the rank of data matrix). It is usually quite low, as we mentioned rarely exceeds ten. The number of clusters thereafter is a matter of arbitrary decision. Several methods exist in the literature to estimate, verify the correct number of clusters^26–28^, all can be used. It is a good practice, however, to keep the number of clusters above (or equal) the number of the significant V_i_ maps.

The presence and number of clusters are very much obvious in the model maps where different species were spatially separated from each other (see Supporting Information 2). Clusters can also be specified in the case of real samples, although the spatial and spectral overlaps make the number, determination, and interpretation of the meaning of the clusters more difficult.

An average spectrum characteristic for the cluster can be calculated (i) from the original spectra of the map points belonging to the cluster or (ii) from the noise filtered reconstructed spectra obtained after inverse SVD calculation, using only the significant (n_e_) elements of the **U**, **W**, and **V**^**T**^ matrices. We have used the first approximation throughout the paper. Differences between cluster spectra may reveal the structural/compositional inhomogeneity of the sample. Comparing the spectra of different clusters may also help in the determination of the number of relevant clusters (identical or very similar spectra are indications, although not a clear-cut rule, of joining the corresponding clusters).

#### Generating model maps

For model maps, usually two different (slightly overlapping) single peak spectra were mixed. Both spectra were a Lorentzian function with different central frequencies (k_i_) and full width half maxima (γ_i_): ***spectrum-1***: k_1_ = 1620 cm^−1^, γ_1_ = 60 cm^−1^; ***spectrum-2***: k_2_ = 1850 cm^−1^, γ_2_ = 40 cm^−1^.

Three different maps were calculated, ‘distinct’, ‘overlapping’ and ‘on top’, according to their relative locations.

In the ‘distinct’ model, the two model spectra were arranged in two distinct concentric circles (*circle-1* and *circle-2*), and their distribution along the circle radius followed a rectangular function (yes or nothing). Thus, the resulting map contained a circle containing only the first, and another circle containing only the second model spectrum.

In the ‘overlapping’ model, the radii of the circles were the same, but the intensity distribution of the model spectra along the circle radius followed a Lorentz function. Therefore, ***spectrum-1*** centered on *circle-1* had a small contribution of ***spectrum-2*** centered at *circle-2* and *vice versa*. All map points contained a mixture of the two model spectra.

In the ‘on top’ model, only one circle (*circle-1*) was used. Both spectra were centered on the same circle and both had the same Lorentz distribution along the circle radius. ***Spectrum-1*** distributed all around *circle-1* in 360°, while ***spectrum-2*** had an additional Lorentz distribution along *circle-1* in an arc with 20° half-width. Detailed explanations and pictures are presented in Supplementary Information 2.

### Raman spectroscopy on real test samples

In the model maps, we have used white noise to simulate the experiments, and we assumed that in real samples the noise is also white noise. Nevertheless, in real experiments the noise is very rarely white (i.e. in Raman spectroscopy the noise usually has a Poisson distribution), therefore the correct way of analyzing experimental data is to transform them to have white noise. There are several methods of how these transformations should be performed^29^, however, in this study, we omitted using them.

#### Dolomite (DOL)

The geological sample was a rock core-sample from the Bm-1 deep borehole (South-West Hungary). The type of the sample was a quartz-carbonate vein in sandstone (Téseny Sandstone Formation). Its analyzed minerals were dolomite (CaMg(CO3)_2_) and ankerite (Ca(Fe_0.6_,Mg_0.3_,Mn_0.1_)(CO_3_)_2_).

DOL Raman map was recorded on a Thermo Scientific DXR Raman Microscope using a 50x objective, with 780 nm excitation of a diode-pumped solid-state (DPSS) laser, at 14 mW laser power, using 830 lines/mm grating, and spectral resolution of 4 cm^−1^. The aperture of the spectrograph was 100 µm.

Raman data collection settings were: Exposure time: 3 s, 5 repetitions at each point. The background was obtained from 512 exposures. Steps between the measuring points were 30 µm both in X and Y directions. The total scanned area was 1560 ×1560 µm, providing altogether 2704 Raman spectra.

#### Biological sample (*Catharanthus roseus*)

Species *of Catharanthus roseus*, a tropical plant commonly known as periwinkle, were grown in a greenhouse. Leaves, free-hand sections of the stem, root were collected and further cut to prepare cryo-blocks. Thin, excised pieces of few mm^2^ plant tissue were transferred into disposable plastic cryomolds (Tissue-Tek®; Sakura Finetek), cryogel (Tissue-Tek® O.C.T. Compound; Sakura Finetek) was added and the blocks were immediately frozen in liquid nitrogen and stored at −80 °C.

For Raman microscopy dissects from leaves, stem, and root within the frozen blocks were cut with a cryostat (Leica CM3050 S, Leica Biosystems) into 4–6 µm thin sections and mounted on SuperFrost microscope slides (Carl Roth GmbH). These samples were kept at room temperature for a couple of minutes and then washed in a series of ethanol solutions: 1 minute in 50% ethanol, 1 minute in 75% ethanol and 5 minutes in 90% ethanol. We added a few µl of MQ water, covered the tissue sections with a cover glass and sealed the samples with nail polish.

Raman micro-spectrometry was performed on an inverse Raman microscope (XploRA® INV, HORIBA Jobin Yvon GmbH) using a Nikon CFI PlanApo 100x/1.4 oil immersion objective. A solid-state laser at 532 nm (40 mW) provided excitation and Raman spectra were recorded with a thermoelectrically cooled EMCCD (DU970P-FI-328, ANDOR Oxford Instruments). Altogether 168,783 spectra were collected from an ~(52.3 μm*45.7 μm) area with 120 nm step size both in X and Y directions.

## Results

### Model maps

#### ‘Distinct model’

In the ‘distinct model’, the two different spectra were completely separated in space arranged in two concentric circles (outer circle: ***spectrum-1***; inner circle ***spectrum-2***), no cross-contamination was present (see Supplementary Information 2). No noise was added.

**Figure 1:**
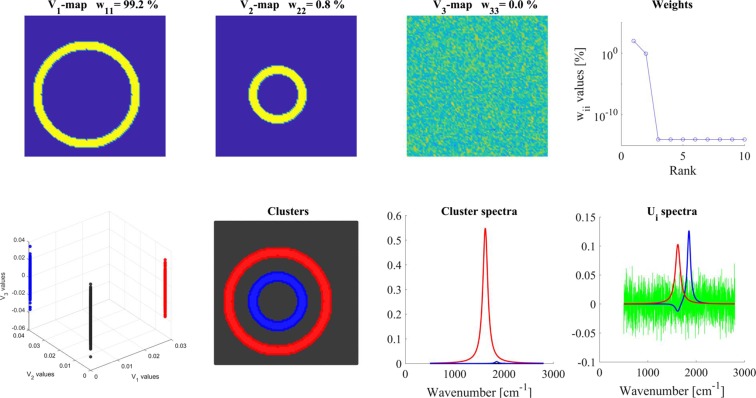
Analysis of the ‘distinct’ model. Different panels are assigned by their titles. Color code for U_i_ spectra: red-U_1_, blue-U_2_, green-U_3_. For clustering, 2 V_i_ maps were used and 3 clusters were calculated. For details, see the text.

The most important results obtained in this simulation are presented in Fig. 1. The ‘average’ V_1_-map showed one ring since the intensity ratio of ***spectrum-1*/*spectrum-2*** was 100:1, thus, only the distribution of ***spectrum-1*** was seen. V_2_-map showed the largest deviation from the average, here the distribution of ***spectrum-2*** became clearly visible. V_3_-map contained only the digital noise. **W** had only two significant W_ii_ values (‘Weights’ panel). As regards the basis-spectra (‘U_i_-spectra’ panel), since U_1_ was the average spectrum of the map the minute contribution of the minor spectrum was only hardly visible. U_2_, however, clearly indicated the deviation from the average at the maximum of ***spectrum-2*** (1850 cm^−1^), and it showed a small negative peak at the maximum of ***spectrum-1*** (1620 cm^−1^).

The V_1_-V_2_-V_3_ scatter map showed three distinct groups; therefore, for clustering, we used the significant V_1_-V_2_ maps and calculated three clusters. The spectra of the clusters were well separated, the blue spectrum (which agreed perfectly with ***spectrum-2***) was 1% of the red spectrum (which agreed with ***spectrum-1***), the background was a straight line (‘Cluster spectra’ panel).

#### ‘Overlapping model’

**Figure 2:**
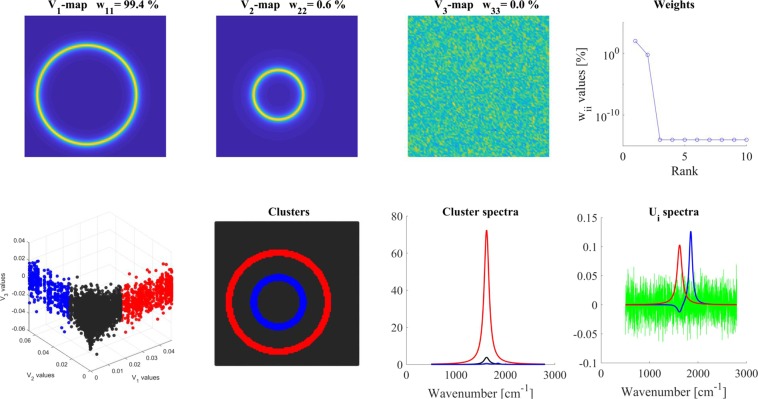
Analysis of ‘overlapping model’. Different panels are assigned by their titles. Color code for U_i_ spectra: red-U_1_, blue-U_2_, green- U_3_. For clustering, 2 V_i_ maps were used and 3 clusters were calculated. For details, see the text.

The overlapping model (Fig. 2) also contained ***spectrum-1*** and ***spectrum-2*** in two concentric circles at a 100:1 intensity ratio, but their spatial distribution was overlapping (see Materials and Methods and SI-2). The V maps indicated that there were two significant components in the map. Conventional analysis, the equivalent of the ‘average’ V_1_-map, displayed only the ***spectrum-1*** distribution and did not show ***spectrum-2*** at the inner circle. The V_2_-map, however, indicated the presence of ***spectrum-2****.* For both spectra, their distributions perpendicular to their respective circles were also visible. The **W** matrix had again two significant values in its diagonal (‘Weights’), The V_1_-V_2_-V_3_ scatter plot, however, did not give a clear indication about how many different clusters were present.

The U_i_ spectra were very similar to those of the ‘distinct’ model; U_1_ displayed the average spectrum, U_2_ the same deviations from the average at two maxima of ***spectrum-1*** (1620 cm^−1^) and ***spectrum-2*** (1850 cm^−1^).

Calculating three clusters by using two components (V_1_ and V_2_ maps); the cluster map (‘Clusters’ panel) exhibited the expected spatial distribution of ***spectrum-1*** and ***spectrum-2***. The cluster spectra, however, were not clear spectra of either ***spectrum-1*** or ***spectrum-2***. Since every map point had a contribution from both spectra, the cluster spectra (including the background spectrum) were also a mixture of the two components spectra. Nevertheless, cluster spectra gave back the real components at the cluster positions. Red cluster spectra almost exclusively contained only ***spectrum-1***, while the blue cluster spectrum was a mixture of the ***spectrum-1*** and ***spectrum-2***. In the blue cluster spectrum, the intensity of ***spectrum-1*** depended on the extent of its overlap with ***spectrum-2***, therefore its contribution (due to its 100 times higher intensity) could be actually higher than that of the ***spectrum-2***.

#### ‘On top’ model

**Figure 3:**
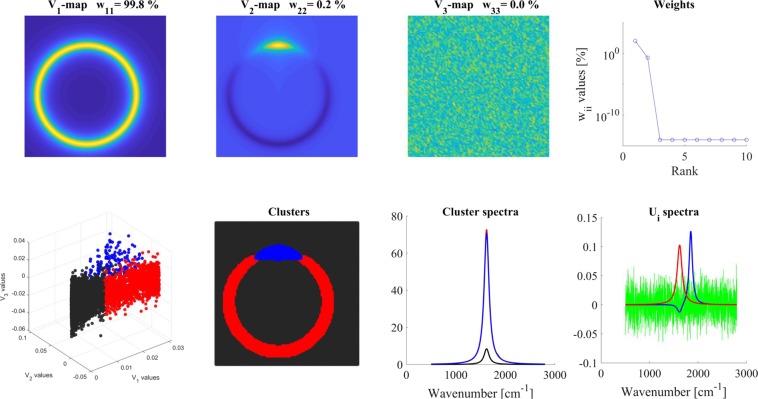
Analysis of ‘On top’ model. Different panels are assigned by their titles. Color code for U_i_ spectra: red-U_1_, blue-U_2_, green- U_3_. For clustering, 2 V_i_ maps were used and 3 clusters were calculated. For details, see the text.

This model (results are shown in Fig. 3) contained a circle of ***spectrum-1*** (1620 cm^−1^) and additionally in form of an arc (half-width was 20°) ***spectrum-2*** (1850 cm^−1^) on the same circle. ***Spectrum-2*** had again only ~1% contribution (for details see Materials and Methods and SI-2). Conventional analysis (the equivalent of V_1_-map) could not reveal the second component here either. V_2_-map indicated again two components and showed their localizations. The **W** matrix values and the U_i_ spectra (‘U_i_-spectra’ panel) were very much similar to the ‘distinct’ and ‘overlapping’ models, but the V_1_-V_2_-V_3_ scatter plot did not give a clear indication about the number of clusters. Nevertheless, based on the significant V_1_ and V_2_-maps, and choosing three clusters, meaningful cluster distribution (agreeing with the expectation) and adequate cluster spectra were obtained. The cluster spectra were again a mixture of the component spectra as it should be. The cluster spectra were dominated by ***spectrum-1*** (1620 cm^−1^). In addition, the blue cluster spectrum showed a small contribution (~1%) of ***spectrum-2*** (1850 cm^−1^). This is because even at those places, where ***spectrum-2*** was present with its maximal intensity, 99% of the signal was coming from ***spectrum-1***.

#### The effect of noise on the analysis

While already plain SVD is an excellent noise-reducing method, in real life, the particular data set might need additional, experiment-optimized filtering, and setting up criteria for adequate noise-filtering. Indeed, there are many different methods to filter noises according to the nature of the particular data set. For Raman spectra, several sophisticated methods were elaborated (e.g.^29,30^, or look for a very good discussion/presentation of the noise-reducing possibilities^31^).

However, here, we limit ourselves only for the demonstration of the theoretical noise tolerance of our method. This, in the case of real samples, can be enhanced by applying specific noise-filtering, adapted to the characteristics of the given sample data prior to the SVD analysis.

**Figure 4:**
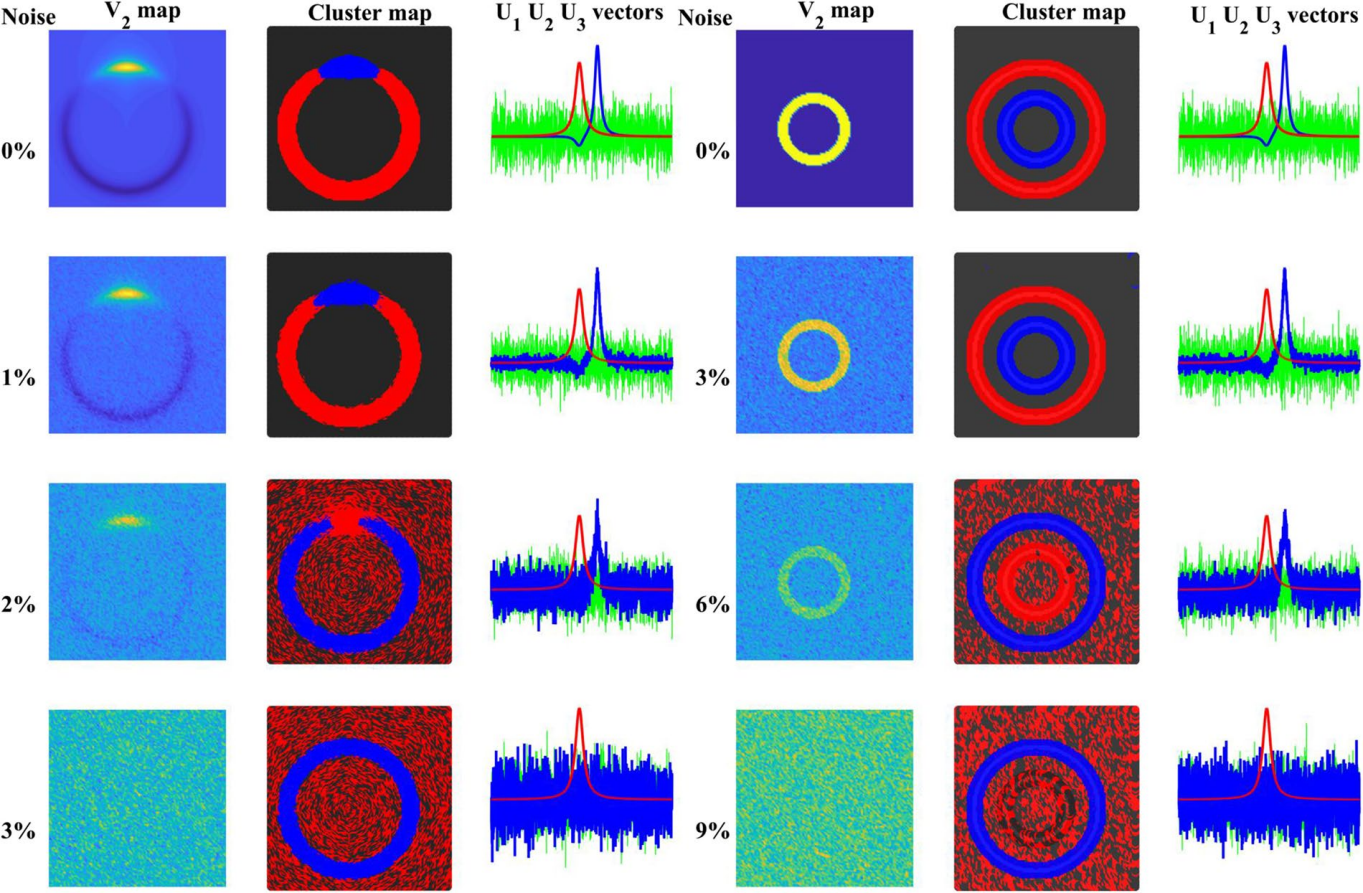
Effect of noise on the SVD-clustering. Left panel: ‘distinct’ model, with 0, 2, 4, 6, 8‚ and 10% noise added to the calculation. Right panel: ‘On top’ model with 0, 1, 2, 3, and 4% noise added. In each panel, the first column shows the V_2_ map, the second column presents the cluster maps and the third column the U_i_ spectra.

To investigate the effect of the noise we added a different amount of white noise to the spectra (Fig. 4). Its intensity was related to the maximal intensity of ***spectrum-1***.

If the two spectra were well separated spatially (‘distinct’ model), the noise had a minute effect. Only as high as 9% noise made ***spectrum-2*** (~1% weight) in *circle-2* unrecognizable in the V_2_-map. The ‘on top’ arrangement was more sensitive to the noise, already 3% of it made the second component (the 20° arc on top of *circle-1*) invisible.

### Geological sample

The composite dolomite and ankerite minerals are (multi-cations) vs. carbonate complexes, whose actual Ca, Fe, Mg, Mn composition may change from location to location and may reflect the evolution of the mineral over a long period of time. Raman spectra are very sensitive to the masses of the atoms participating in a vibrational mode and to the strength of the interaction between them. Therefore, demonstrating minor Raman band frequency shifts due to spatial changes in the relative amounts of the metal ions can be a good test of the sensitivity of the present data analysis, and the frequency shift in itself also has a structural interest.

**Figure 5:**
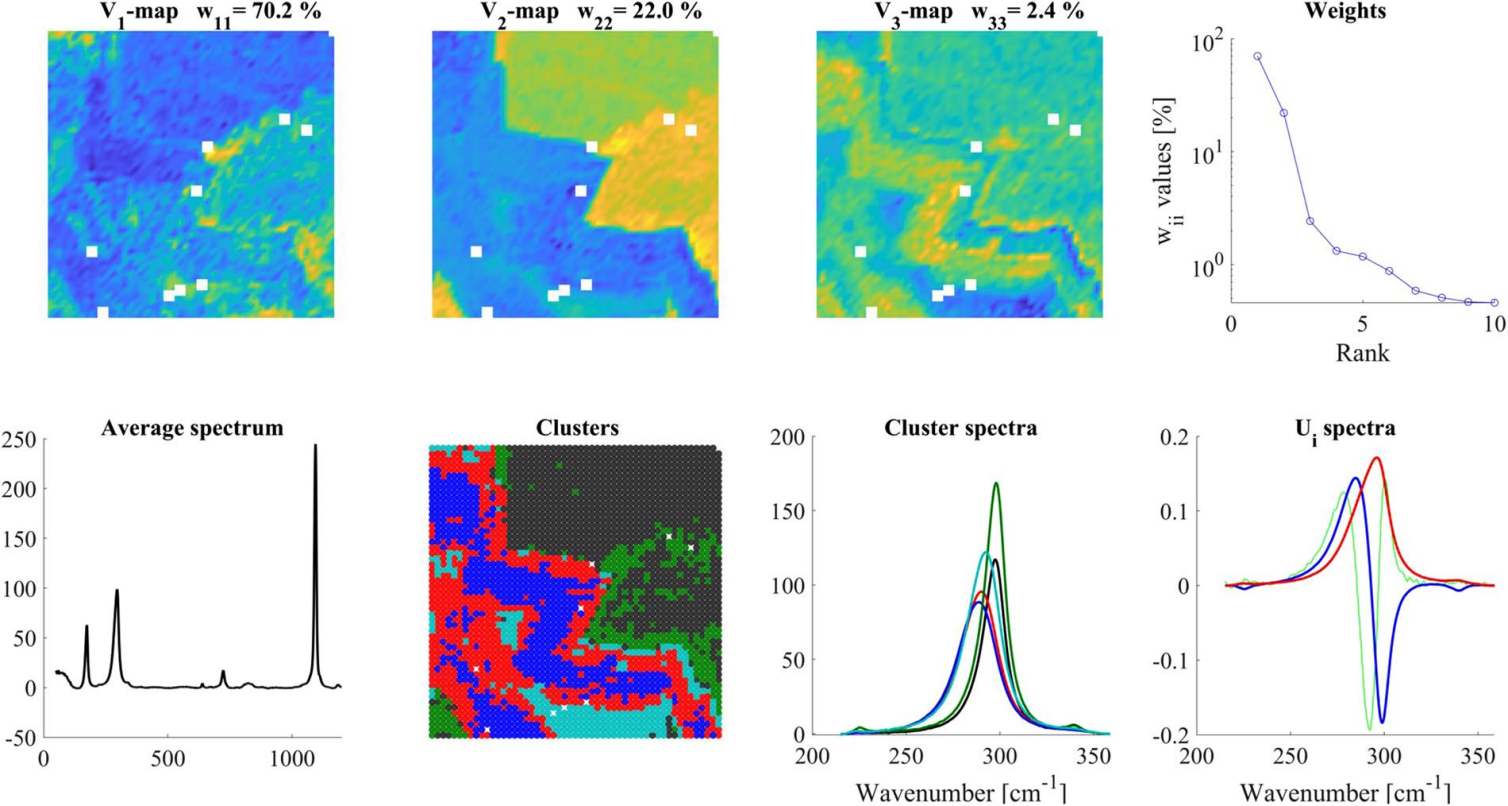
The Raman spectrum of the dolomite (DOL) sample (Average spectrum). Other panels - SVD analysis of the DOL Raman spectra in the 215–359 cm^−1^ region. Color code for U_i_ spectra: red-U_1_, blue-U_2_, green-U_3_. For clustering, 3 V_i_ maps were used and 5 clusters were calculated.

The average of altogether 2704 DOL Raman spectra was plotted in Fig. 5 (‘Average spectrum’ panel). The spectrum was a relatively simple Raman spectrum, exhibiting four considerable Raman bands^32,33^, from which the behaviors of only the bands at 296 and 1095 cm^−1^ are discussed in the present paper.

SVD-cluster analysis of the spectrum around the 296 cm^−1^ band is shown in Fig. 5. According to the W_ii_ values (‘Weights’ panel), the effective rank - n_e_ was around either 3–4 or 5–7 in this experiment depending on the chosen limit. This real sample shows well the dilemma of choosing the proper n_e_ value. Looking at the higher ranking V_i_-maps, we found hardly any structure from V_5_, no structure at all from V_7_ (data not shown), while on the basis of the slowly decreasing W_ii_ values one might assume more significant components.

For simplicity, to remain in harmony with the other discussions, we considered only V_1_-V_3_-maps (minimal amount of information was lost, which did not affect the conclusions drawn about the DOL sample).

We assumed five clusters and calculated their corresponding cluster spectra (‘Clusters’ & ‘Cluster spectra’ panels). In the SVD analysis, the largest deviation from average was a downshift of the band (U_2_ basis-spectrum). Accordingly, the red, cyan and blue cluster spectra were explicitly downshifted as compared to the others. The corresponding red and blue clusters spread diagonally on the lower left part of the map (Fig. 5, Clusters). The red clusters were at the edges of the domains, which in their interior contained the blue clusters. Red and blue cluster spectra are very similar to each other.

The band at around 296 cm^−1^ is an external deformation vibration mode of the crystal lattice originating from the librational movement (E_g_, L) between the cations (Ca, Mg, Fe, Mn) and the carbonate ion^34,35^. Since the Ca content in dolomite and ankerite is almost identical, the frequency shifts of this band can not originate from occasionally changing Ca content. In this respect the relative amounts of Fe and Mn ions, that replacing Mg, are important. The more Mg ions are replaced the lower the frequency of the band^36^. This replacement evidently involves ankerite, thus one can expect frequency shifts of the 296 cm^−1^ band in the ankerite domains of the mineral. The observed downshift may indicate a minor difference in the composition of the ankerite at the edges of its domains, probably due to the changing external cation-content over time, and what we see is the result of a slow penetration of the newly arriving cation(s) into the crystals.

**Figure 6:**
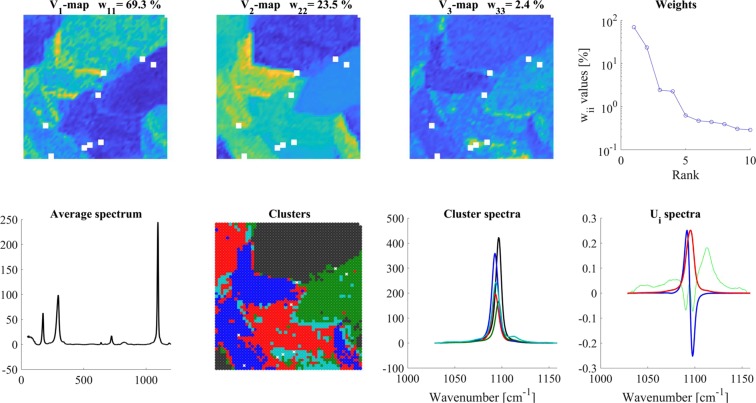
SVD analysis of DOL Raman spectra in the 1028–1159 cm^−1^ region. Color code for U_i_ spectra: red-U_1_, blue-U_2_, green- U_3_. For clustering 3 V_i_ maps were used and 5 clusters were calculated. White squares on the maps indicate pixels from where “wrong” spectra (due to extreme noise resulting out-of-range points in the scatter plots) had to be eliminated.

SVD-clustering of the 1028–1159 cm^−1^ region is presented in Fig. 6. In this case, also three V_i_ maps were taken into account and five clusters were calculated. U_1_ (red) basis-spectrum (the average) was centered at 1095 cm^−1^. The U_2_ basis-spectrum (blue) showed a downshift. Blue, red and cyan cluster spectra exhibited similar downshift and their corresponding clusters were located on the left-bottom part of the map. Black and green cluster spectra displayed the measured 1095 cm^−1^ band.

The band at around 1095 cm^−1^ is due to the symmetric stretching vibration of the CO_3_ ion (A_1_g, v_1_CO_3_)^37^. The in-phase oscillation of the three oxygen atoms is coupled to the movement of the central carbon atom, which will depend on the type of the cations attached to its other side.

Since the strength of the interaction between the CO_3_ ion and cations depends on their distances, which are determined by their ion-radii, and which are different for different cations (Mn (0.80 Å) > Fe (0.76 Å) > Mg (0.65 Å)), changes in their relative amounts will affect the v_1_CO_3_ frequency^38^. The smaller the ion-radius the stronger the interaction, i.e. higher is the v_1_CO_3_ frequency. For pure dolomite (CaMg(CO_3_)_2_) it is 1099 cm^−1^, for pure ankerite (Ca(Fe_0.6_, Mg_0.3_, Mn_0.1_)(CO_3_)_2_) it is 1093 cm^−1^ ^36^.

This may mean that in the different domains higher or lower dolomite/ankerite ratios were present since the pure ankerite/dolomite v_1_CO_3_ frequencies are at 1093/1099 cm^−1^. Considering the signs of the U_2_ spectrum (positive around 1093 and negative around 1099 cm^−1^), that means that on the V_2_-map, the domains in yellow/green color either contain higher ankerite/dolomite ratios as compared to the domains of blue color, or they represent ankerite with different cation composition. If the U_1_ basis-spectrum was a single sharp band evidently coming from one single vibrational mode, and the U_2_ spectrum was a clear shift of the same band, we can afford such a direct explanation. Clustering reassuringly agrees very well with the domains seen in the V_2_-V_3_-maps (Fig. 6) if comparing the location of the red and blue vs. black and green clusters and their corresponding spectra.

Impressive proof of the sensitivity of the data analysis and clustering is the difference between the clusters of the 296 cm^−1^ and the 1095 cm^−1^ bands. While both vibrational modes are changing due to the same cation exchange, the 296 cm^−1^ lattice deformation mode is directly affected, but the 1095 cm^−1^ carbonate stretching is only indirectly affected by the Mg ← (Mn, Fe) replacement in the cation ↔ carbon bond. This is visible in the much more detailed cluster map of the 296 cm^−1^ bands.

### Biological sample

As a biological example, the fixed cross-section of *Catharanthus roseus* root was chosen. We specifically focused on the xylem component of the organ. This sample area is of special interest in ongoing projects but, in this study, it merely serves for demonstration. In flowering plants, xylem comprises four fundamental types of cells: tracheids, vessels, xylem fibers, and xylem parenchyma, the latter one being the only living component in the xylem with distinctive nucleus and cytoplasm. These tissue elements not only differ in shape and in cell organelle composition but also show characteristic cell wall structure. Xylem fibers, for example, usually have a very thick lignified secondary cell wall which is missing in parenchyma cells and in gelatinous fibres. Vessel cells or vessel elements together with tracheids also contain lignin which - apart from their primary role of conducting water, minerals and other nutrients, - provides mechanical support. Chemical characterization of cell walls in specific plant tissues has been a major application of Raman imaging for decades now^39,40^.

Depending on cell type and tissue three/four types of major polymers with distinct Raman spectra constituted the plant cell walls in various percentages. These polymers were: cellulose, hemicellulose, lignin and pectin^39,40^.

**Figure 7:**
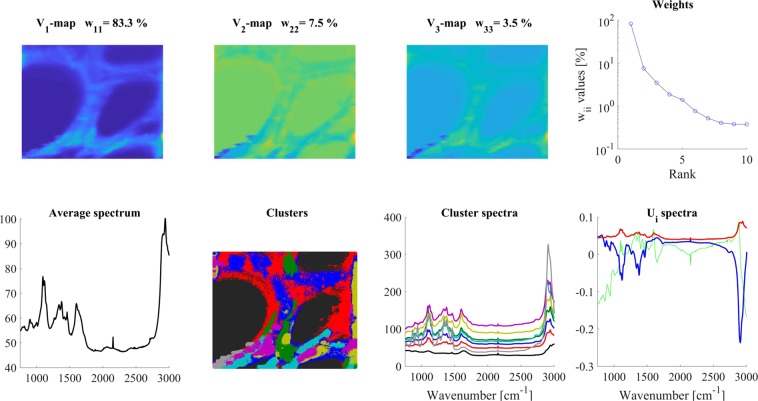
SVD analysis of a *Catharanthus roseus* root cross section, 40–3400 cm^−1^ region. Note, the clearly visible cell walls in V_1_-map, and that W plot indicates meaningful components up to W_77_. Color code for U_i_ spectra: red-U_1_, blue-U_2_, green- U_3_. For clustering 6 V_i_ maps were used and 8 clusters were calculated.

The average Raman spectrum representing a random area in periwinkle root xylem is shown in Fig. 7 (‘Average spectrum’ panel). The fingerprint region (1000–1800 cm^−1^) of the spectrum is rich in bands, and there was also a strong composite band in the 2700–3100 cm^−1^ region due to different C-H stretching vibrations. Upon former studies, integrating the signal over these regions, an overview of the cell wall structure became visible, in which several polymers were present, and their individual contribution varied considerably^41,42^. The spatial distribution of the various polymers could also be visualized by integrating the Raman signal over certain bands assigned predominantly to a given compound.

We performed SVD analysis and clustering of the Raman spectra obtained from mapping the root section. From the results shown in Fig. 7, one may conclude that the whole Raman spectra (50–3200 cm^−1^) are too complex for a detailed analysis. They contain many significant components; six significant V_i_ maps were used for clustering (only V_1_-V_2_-V_3_ maps are shown in Fig. 7) and eight clusters were calculated, although probably there are many more present (eight cluster spectra is the upper limit in our present software). Nevertheless, the cluster map clearly indicated distinct regions in the cell walls (Fig. 7. ‘Clusters’ panel).

We have chosen the 1000–1200 cm^−1^ region as characteristic for cellulose^43^, and the 1550–1700 cm^−1^ region, dominated by phenyl groups, for lignin^39,44^. Comparing the maps of these regions, one can get an impression of the structural inhomogeneity of the sample. (For both regions, a linear baseline was subtracted before the analysis).

**Figure 8:**
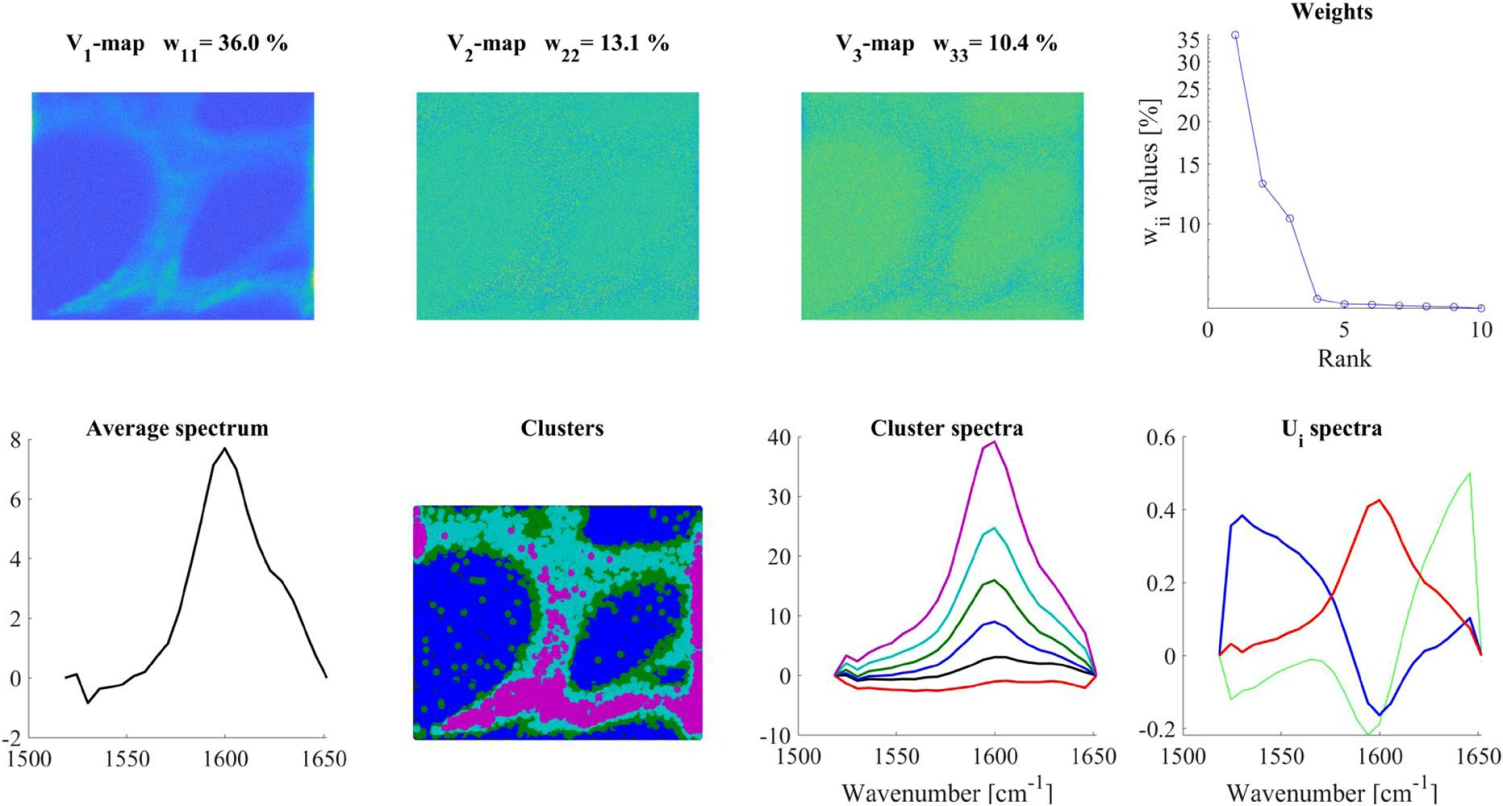
SVD analysis of the 1519–1652 cm^−1^ region (characteristic for lignin) of a *Catharanthus roseus* root gross section. Color code for U_i_ spectra: red-U_1_, blue-U_2_, green- U_3_. For clustering, the V_1_ map was used and 6 clusters were calculated.

The region characteristic for lignin (1550–1700 cm^−1^, Fig. 8), while it had a V_1_-map similar to that of the whole spectrum, already its V_2_-V_3_-maps exhibited weakly any structure. The W_ii_ values could allow a higher n_e_ value (up to 3–5), but based on visual inspection, we calculated the cluster spectra only from the V_1_-map and assumed six clusters. The number of clusters (an arbitrary decision) was based on the iterative visual inspection of the obtained cluster maps (Fig. 8 ‘Clusters’ panel). It can be seen from the cluster spectra (Fig. 8 ‘Cluster spectra’ panel) that the black and the red clusters form the background, there is an interfacial cluster between the walls and the lumen of the cells, and there are two definite regions of different lignin structure in the cell walls.

Here, it should be noted that using only the V_1_-map for clustering means that only the intensity differences of the individual spectra were considered (like in a conventional case). The U_2_-U_3_ basis spectra indicate also predominantly intensity changes within the sample since their shapes are very similar. Spectral variations between the U_i_-spectra depend also on the sample. E.g. in the case of Raman spectra, if the studied molecule has a definite structure, which is not changing among different conditions, then the U_i_-spectra can reflect only relative intensity changes. If the studied molecule (e.g. a protein or a lipid) can have very different structures at different points of the sample, the U_i_-spectra will reflect spectral differences. For lignin, evidently, the first situation applies, but as we can see below, cellulose has higher structural variability (expressed by a higher number of significant V_i_-maps, and spectrally more different U_i_-spectra) in the same sample.

**Figure 9:**
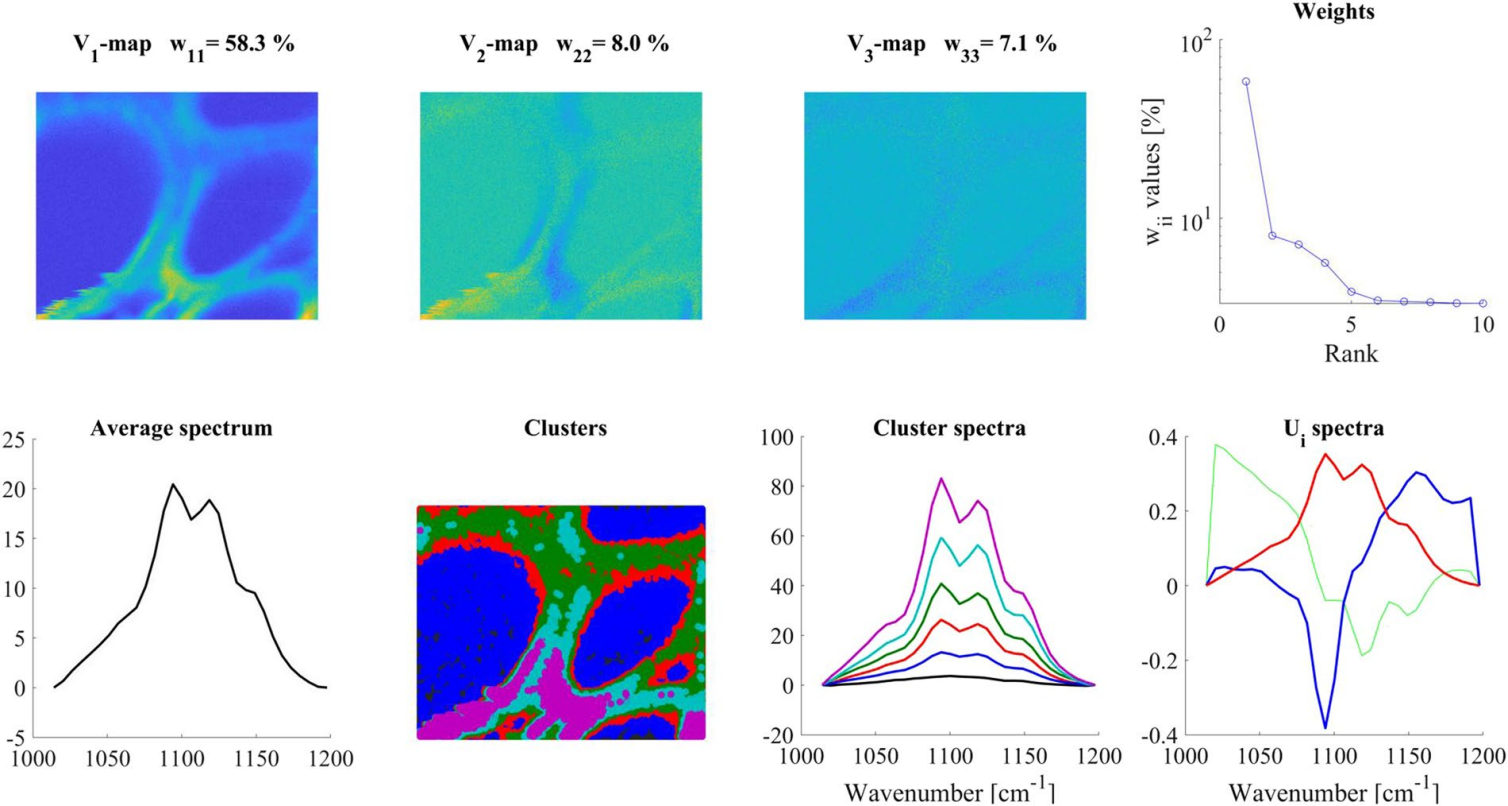
SVD analysis of the mostly cellulose-related 1000–1200 cm^−1^ region of the Raman spectra recorded on the cross-section of a *Catharanthus roseus* root. Color code for U_i_ spectra: red-U_1_, blue-U_2_, green- U_3_. For details, see the text. For clustering, the V_1_ map was used and 6 clusters were calculated.

In the 1000–1200 cm^−1^ (cellulose) region, there were three significant V_i_-maps (Fig. 9). The V_1_-map is similar, but V_2_-V_3_-maps were very different from those obtained for the phenyl group region (1550–1700 cm^−1^). For clustering, only the V_1_ map was used and six clusters were calculated. The cluster areas had sharp boundaries, and, according to the cluster distribution, the walls between the cells were made up of differently structured celluloses. The component bands within the cluster spectra exhibited different relative intensities, which might reflect differently structured celluloses in the different parts of the cell wall. These structural differences were very similar to those found for example between microcrystalline and amorphous (apple wall) celluloses^43^, where the relative intensities of the 1157,1120, and 1095 cm^−1^ bands were changing depending on the cellulose form. Going into the details of the cellulose conformation, orientation with regard to the polarization of the exciting laser light^42,44–46^ goes beyond the scope of the present demonstration, but it is clear that subtle differences can be revealed and thoroughly discussed in a focused study.

## Discussion

It is always a challenge to evaluate an image. The main question is: what kind of information can be extracted from the presented data. Efforts have been made to separate different cell components, organelles or distinguishing various types of cells in tissues or *in vitro* cultures, visualize and analyze spatially separated but blurred crystals, to follow the time-dependent changes in CT or MRI, etc.

The main use of SVD in image analysis was noise filtering so far. We extended SVD with a clustering method, using the significant rows from the V^T^ matrix as coordinates of image points in a n_e_-dimensional space. This way every image point had a corresponding point in the n_e_-dimensional space and formed a point set. Clustering was applied to this point set.

A similar method was previously published^24,25^ using PCA scores for clustering. PCA-clustering is giving suitable spatial resolution only in the case when the sample contains spatially distinct components (i.e. our ‘distinct’ model), while SVD-clustering can determine the structure in the overlapping samples as well. A detailed discussion and comparison of the two methods are given in Supplementary Information 4.

### Reliability, robustness, and use of the SVD-clustering

We challenged the method with artificially fabricated maps. The main question was that what kind of information is possible to retrieve from the map using our new method; what structural and spectral information can be gathered. It became clear that if the different components were spatially distinct, our combined method (SVD and clustering) could reconstruct the component spectra. The situation was more complicated when the components were not strictly separated spatially. In these cases, every map point had a contribution from all components; therefore, the cluster spectra were also mixtures of the two artificially fabricated component spectra. The ratio of mixture strongly depended on the extent of the mutual overlap. If the overlap is minute (‘overlapping model’) the cluster spectra can be very different (see ‘Clusters’ panel in Fig. 2), nevertheless, the main component was dominating the cluster spectra even in this case. If the two components were really on top of each other the corresponding cluster spectra were almost identical, only a small contribution of the second component was visible in one of the cluster spectra (see ‘Clusters’ panel in Fig. 3). This was due to the comparable intensity of the major component and it coincided with the minor component. In real samples, this is the most frequent situation. The fact that in this case clustering cannot reconstruct the pure component spectra is a very important conclusion for all clustering methods. This is a frequent “dream” of the data analyses, but here we demonstrated that this is impossible for spatially and spectrally overlapping components.

Nevertheless, several methods in the literature promise to de-convolute to ‘real’ component spectra from the map. Such methods are multivariate self-modeling methods like MCR-ALS^7–11^, SIMPLISMA^6^, OPA^5^ or HIA^15,16^, but many others also exist. All these methods are using an iteration procedure to de-convolute the large set of different spectra obtained at spatial points of the image. The main idea behind these approaches is, that since the ratio of different components is different at every map point, an iteration method can separate the component spectra.

We tried the MCR-ALS (https://mcrals.wordpress.com/download/mcr-als-2-0-toolbox/) to de-convolute a more complicated ‘Triple on top’ model (details and results are presented in Supplementary Information 3). It became clear that multivariate methods (at least MCR-ALS) – similarly to our method - were not able to de-convolute image data into real component spectra. Nevertheless, our method was able to visualize very precisely the structures on the map, despite the very small changes in the map-point spectra, while MCR-ALS failed in this respect as well.

It is our conviction that the impossibility to reconstruct pure component spectra is an inherent characteristic in most of the real experiments. The spectra obtained by any clustering, deconvolution, etc. method will always be mixtures of the component spectra; except if the components are well separated spatially and it is possible to measure pure, real spectra of the components at different points of the sample. Another basis for reconstructing component spectra can be if characteristic features of the components are spectrally well separated. In this case, different spectral regions may refer to different component distributions and this may make the resolution of the component spectra possible. One should consider these issues when regarding published cluster spectra in the literature. If the external parameter does not depict a spectrum, but something different (e.g. time dependence like in the case of CT and MR imaging), the situation is even more complicated.

A further advantage of the SVD-cluster analysis is that the orthonormal U_i_ spectra give information about the real spectral changes, which in the cluster spectra, might be hardly visible. Looking at the U_i_ spectra of all model maps, it is apparent that the corresponding spectra are almost identical in every case. U_1_ (the average spectrum), is dominated by the major component, but U_2_ clearly indicates the presence of the minor component spectrum in every case. In addition, both in the geological sample and in *Catharanthus roseus* the structural changes reflected by the U_i_ basis spectra could be rationalized in the context of the sample.

Custer spectra, obtained after SVD-clustering, provide real information about the spatial distribution of even spectrally slightly different domains of the sample. It is clearly seen that in every case the cluster map gives back the information about the spatial distribution of the different domains (having different component compositions) both in model maps and in geological and biological samples. A reliable representation could be gathered even if the component is hardly visible when using a conventional evaluation method. If there was no noise, a 1% intensity contribution to the map is clearly observable. If we add noise, the 1% intensity contribution is observable even if the noise is 3%. If the components are spatially separated the noise can be even 10 times higher than the effect, the phenomena still remain observable. To our knowledge, no other evaluation method is able to reproduce this.

Therefore, we are confident about the cluster elements even in the case of real samples. Clusters, determined by our method clearly depicted the different spatial regions where different constituents of dolomite/ankerite or cellulose/lignin were present.

### How many components should be considered?

It is always a question when evaluating a map, which is the real number of reliably distinguishable components in the sample. There is no clear-cut answer to this question. De-convoluting methods always face this problem; it strongly depends on the sample, on the noise of the measurement, and also on the external parameter (i.e. spectral) region to be evaluated.

For the whole analysis presented here, it is crucial to determine the effective rank and the “correct” number of clusters. There are several mathematically established methods exist to determine these numbers^15,16,26–28,30^, nevertheless, every one of them contains an arbitrarily chosen factor in order to verify the choice. This work is not devoted to select the best one or to suggest a new one. We doubt that a proper mathematical method would exist which would correctly determine, without any arbitrary parameter, the correct number of rank or the correct number of clusters. When using such a method, the problem of the arbitrariness is simply shifted.

All the methods we tried for rank determination, with the “arbitrary” parameter, suggested in the literature, either over- or underestimated the effective rank of our model images depending on the noise in the simulation. We present a comparative study in Supplementary Information 5 on a model map (‘triple on top’ model) to show how different methods would estimate the “real” number of components of the SVD analysis. A similar study might be done (not shown) comparing the “real” number of clusters as well. For determination of rank, without using complicated mathematical procedures, we utilize the maps made from the V_i_ vectors until the last V_i_ map showing any structure (although we have to admit, that “structure” has no definite meaning).

**Figure 10:**
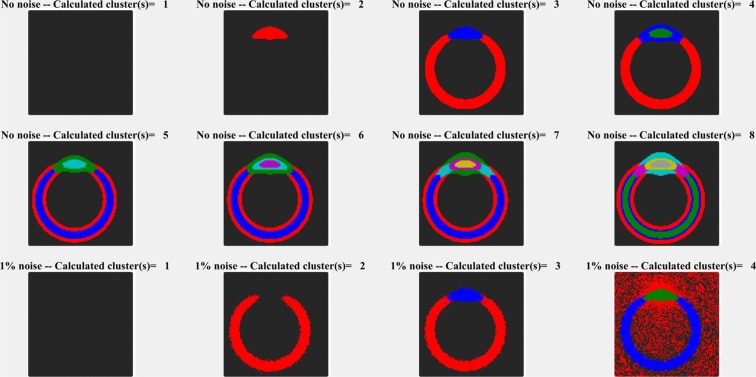
Effect of noise on clustering. The upper two rows represent different clusters in the case where no noise was introduced in calculation. Every cluster can be attributed to a meaningful structural element. The bottom row represent the case when 1% noise was added. In the case of 4 cluster calculation, the red and black clusters became diffuse, indicating that no new structural element was introduced by the 4th cluster.

The number of clusters is not a well-defined number either since clustering is not an unequivocal method. Therefore, it is at the users’ decision, how many clusters are calculated, although there are some indicators worth considering. If the noise is small, a larger number of clusters can be calculated (Fig. 10 upper two rows). In the ‘no noise’ model, every cluster can be interpreted as a different structural element, although the building blocks (the components) are actually the same (there were only two main components in the model maps), only the ratio of the components is slightly different in the different clusters. It means that the number of (chemical) components and the number of structural elements is different in many of the maps. A structural element can have the same or very similar chemical composition, nevertheless, they might be structurally different even if there is just a small deviation in the composition. While a cluster spectrum contains contributions from several components of the sample, the distribution of a cluster spectrum describes a definite, spatial structure, which is (in addition) chemically uniform.

A similar phenomenon was observed for *Catharanthus roseus* when the whole spectrum was included in the evaluation (Fig. 7). The clusters were definite and separate, clear structural elements were portrayed on the cluster map.

In the case of a model map, the introduction of 1% noise (it is the same order of magnitude as the minor component), the increase of the number of clusters beyond a certain value does not add a new structural element to the picture (Fig. 10, bottom row). Instead, the already existing clusters would divide into sub-clusters, cluster map becomes diffuse, and that can easily be recognized from the picture (see the background in the bottom row of Fig. 10). If this happens, the clustering should be stopped. Calculating the compactness of the clusters^26–28^ might also help to determine this point. This was the case of the *Catharanthus roseus* lignin region (Fig. 8) where additional clusters made the picture diffuse, and no additional information could be gathered when the number of clusters was increased. (The effect of limitation in cluster numbers can be seen from the very similar red and black cluster spectra, which are practically only backgrounds. Therefore, their clusters were not visible in the cluster map (Fig. 8, panel ‘Clusters’) because of being ‘covered’ by ‘real’ clusters).

## Conclusions

It has been shown that going beyond the noise-filtering use, Singular Value Decomposition (SVD) can be used for much more detailed analyses as so far.

The SVD amplitude vectors (V_1_…V_i_) were introduced into imaging. Since each V_i_ vector contains all points of the map, plotting a V_i_ vector along the X, Y, Z dimensions of the map reconstructs the spatial distribution of the corresponding U_i_ basis-spectrum. Thus, the average distribution of the measured spectra and the first, second, etc. order deviations from this average can be visualized over the sample.

We introduced a new clustering method using the V_i_ values as coordinates in a n_e_-dimensional space. Clusters were formed in this n_e_-dimensional space by applying any clustering algorytm.

The SVD-clustering analysis is universal; it can be applied to any measurement where data are recorded as a function of an external parameter (time, space, temperature, concentration, species, etc.). Consequently, our method is not restricted to spectral imaging, it can be applied very flexibly since SVD as a non-supervised factoring tool does not require any *a priori* assumption about the data.

Theoretical possibilities and limitations of the SVD-clustering were shown on models, especially in the context of creating, meaning/interpreting of cluster spectra. The most important conclusion is that while clear clustering is possible along compositionally minute differences, the cluster spectra never correspond to a pure spectrum of a sample component, except if the component is spatially perfectly distinct.

To prove the capacities of the SVD-clustering method, results on two real-world examples are shown demonstrating its unique capabilities. In these samples, minute alterations, e. g. changing cation ratios in minerals, or differently structured cellulose and other cell wall polymers in plant root could be spotted and resolved spatially.

## Supplementary information

Supplementary information

## Data Availability

The datasets generated and analyzed during the current study are available from the corresponding author on request.
