# Supplementary material for: SVD-clustering, a general image-analyzing method explained and demonstrated on model and Raman micro-spectroscopic maps
Source: Sci Rep. 2020 Mar 6;10:4238. doi: 10.1038/s41598-020-61206-9 (PMC7060257; doi:10.1038/s41598-020-61206-9)
Supplement: Supplementary file 1 — Supplementary information [file 41598_2020_61206_MOESM1_ESM.pdf]

SVD-clustering, a general image-analyzing method explained and demonstrated on model and Raman micro-spectroscopic maps

Szalontai, B.[1], Debreczeny, M.[2], Fintor, K.[3], and Bagyinka, Cs.[1]

Supplementary Information 1

SVD analysis of maps

When investigating an image (either 2D or 3D) very often special properties can be attributed to every point of the image. The special property can be a spectrum (like Raman or infrared, etc. spectrum), a lifetime curve of a fluorescence decay, adhesion force dependence on the distance (measured on an AFM image), simply time, temperature, and many others. It may happen that not just one but several properties can be attributed (i.e.

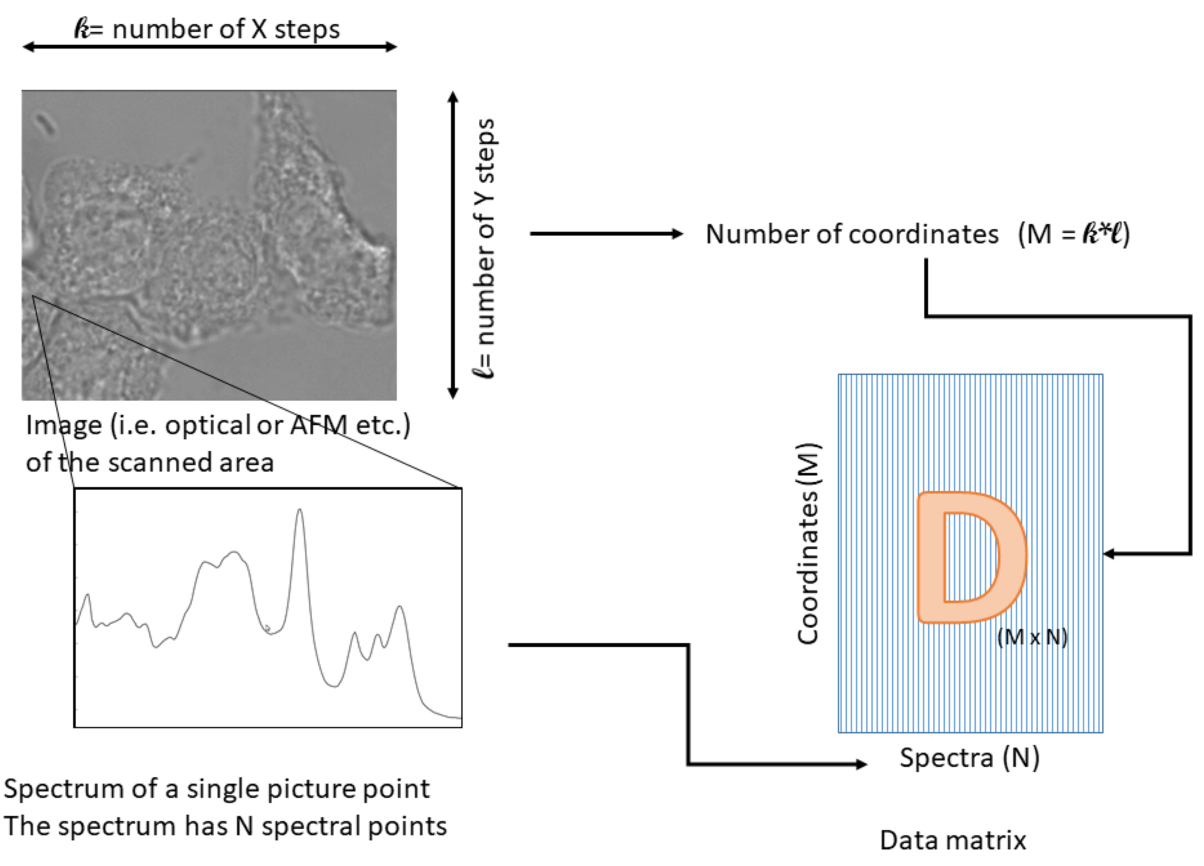

Figure (SI-1) 1. Construction of the data matrix from a 2D Raman micro-image measurement

temperature-dependent Raman micro-imaging) to the same image point. All these cases can be evaluated by SVD (or PCA) analysis. For didactical purposes, in the present study, we consider only 2D imaging and a single property but the presented evaluation method can be used among the most general conditions (as listed above) as well. From now on (because our examples will be from Raman micro-imaging), we will refer to the property attributed to a single image point as a spectrum. We also have to mention that SVD and PCA are the same in this respect; both give exactly the same result [1]. We prefer SVD because it is simpler, easier to understand and calculate (very often PCA is calculated using the SVD) and the algorithm is faster.

The first step for SVD analysis is constructing the data matrix. The steps necessary to construct the data matrix are given in Figure (SI-1) 1. The data matrix is an N*M matrix, where N is the number of data points of the measured spectra, while M is the overall number of picture points.

SVD analysis of a matrix is a well-known mathematical tool in order to factorize a matrix [2]. The result is an arithmetical product of three matrices.

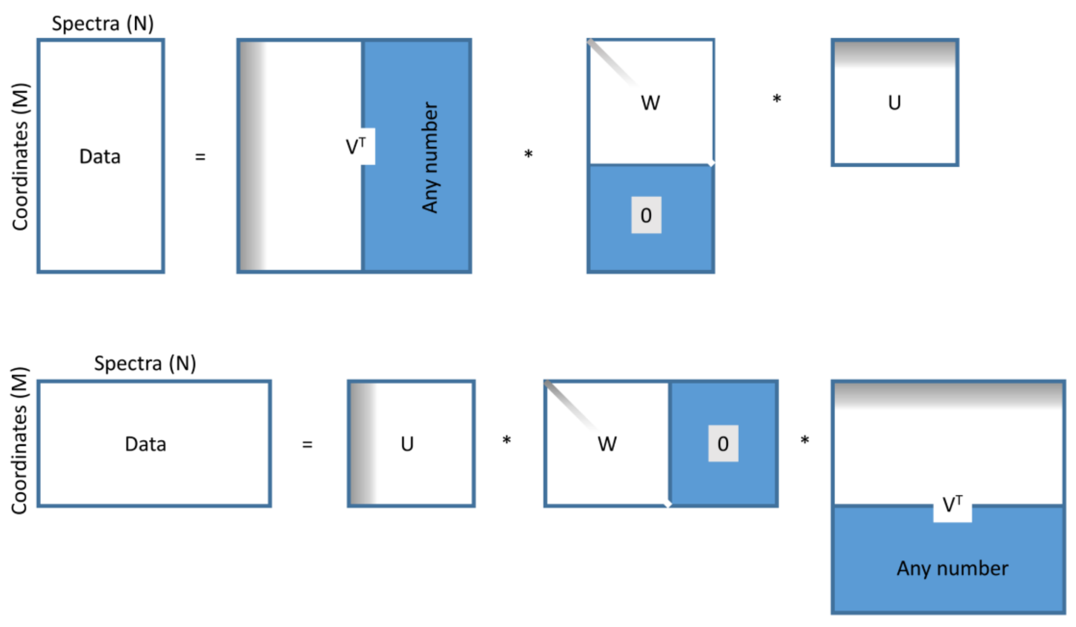

Figure (SI-1) 2. Schematic representation of reduced SVD analysis. Dark areas of U, W and V^T matrices indicate the significant elements of the matrices. Note that depending on the row/column ratios of the Data matrix, U and V^T matrices are switched.

$$\mathbf{D=U * W * V^T},$$

where **U** and **V** are orthonormal singular vectors, and **W** contains - in the diagonal elements – the singular values in decreasing order. If the **D** data matrix contains real measurements (e.g. Raman spectra), the W_{ii} values are decreasing quite fast. It means that only a few of the singular vectors (usually less than 10) are significant (represented as a dark blue region in Figure (SI-1) 2) all the remaining part of the singular vectors can be considered as noise. The product can be reordered (Figure (SI-1) 3) to a sum of N dyadic vector product, multiplied by the weight. The first member of the sum gives the main contribution to the data matrix (D), and so on in decreasing order. Not significant contributions can be neglected. This also can be used as a noise filtering of the raw data.

Since every element of the V_i vector corresponds to a coordinate of the original picture (Figure (SI-1) 1), it is possible to construct a map from a single V_i vector. V_1 will depict the contribution of U_1 to the picture, V_2 will depict the contribution of U_2, etc. The corresponding W_{ii} values describe the weight of the contribution. We used $W_{ii}/\Sigma W_{ii}$ to calculate the percentage of contribution for each spectrum and map.

In real measurements, V_1-map gives an average picture of the sample. It is very similar to the conventional evaluation of the Raman maps where the integral of the spectrum (or spectrum part) is used since U_1 can usually be considered as the 'average' of the spectra. V_2 will show us the first-order deviation from the 'average', V_3 the second-order deviation, etc.

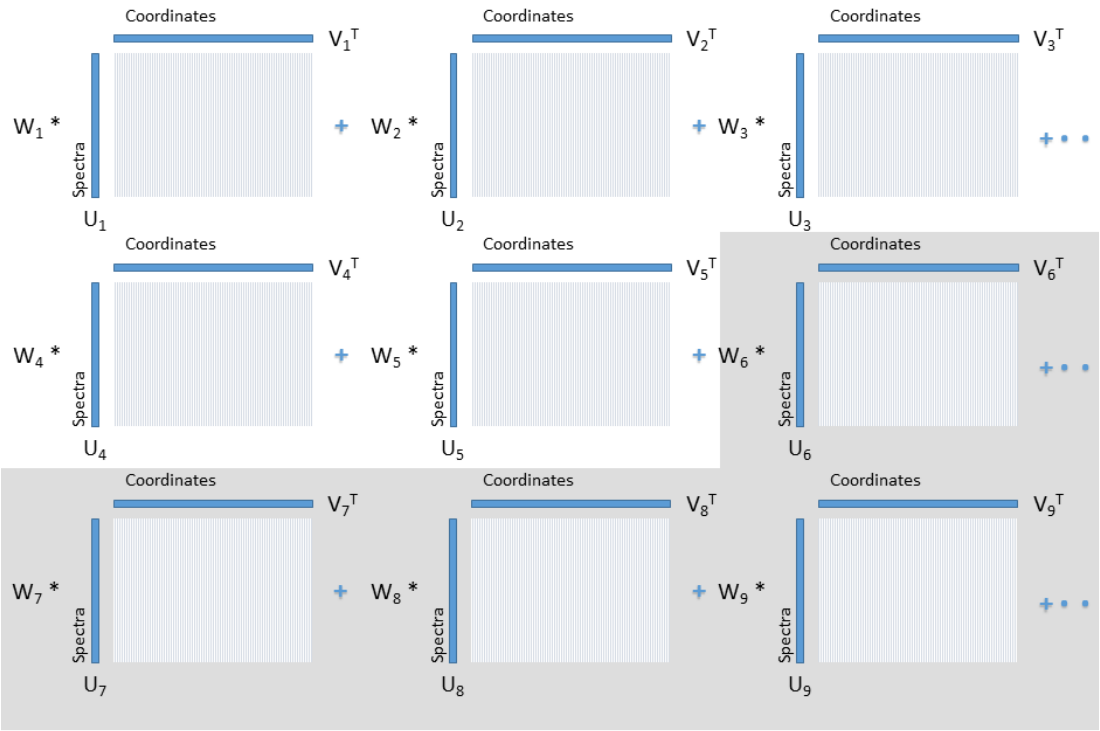

*Figure (SI-1) 3. Dyadic vector products of the component matrices as reordered after SVD calculation. As an example, here, the significant number of components (**n_e**) were chosen being five (n_e=5). Non-significant matrices are dimmed.*

These deviations have a smaller and smaller contribution to the overall picture.

Supplementary Information 2
Fabrication of model maps

Three different maps were fabricated. In every map, two different (slightly overlapping) spectra were mixed with different spatial distribution. Each spectrum contained a single peak. The three different maps were, ‘distinct’, ‘overlapping’, and ‘on top’ model maps (see Figure (SI-2) 1). For the ‘distinct’ model we generated two concentric circle areas with arbitrary width, one for each spectrum (the distribution of the spectra followed a rectangular function along the circle radius). If the mapping point was located within the circle area, the first or the second spectrum was assigned to the point, respectively with maximum intensity. The resulting map contained a circle with the first spectrum and another circle with the second spectrum. In the ‘overlapping’ model the same spatial arrangement was used, but instead of the rectangular distribution of the circles, a Lorentz function was used along the circle radius as a scaling factor for calculating the distribution to the map point of the corresponding spectrum. Therefore, ***spectrum-1*** had a small contribution to the maximal contribution of ***spectrum-2*** and *vice versa*. All map points contained a mixture of the two spectra. In the case of ‘on top’ model, only one circle and Lorentz distribution were used. The second spectrum was distributed on the top of the first one it had an additional Lorentz distribution with 20° half-width along the circle. The resulting map had a circle of ***spectrum-1*** and on the north part of the circle an additional, extra contribution from ***spectrum-2***.

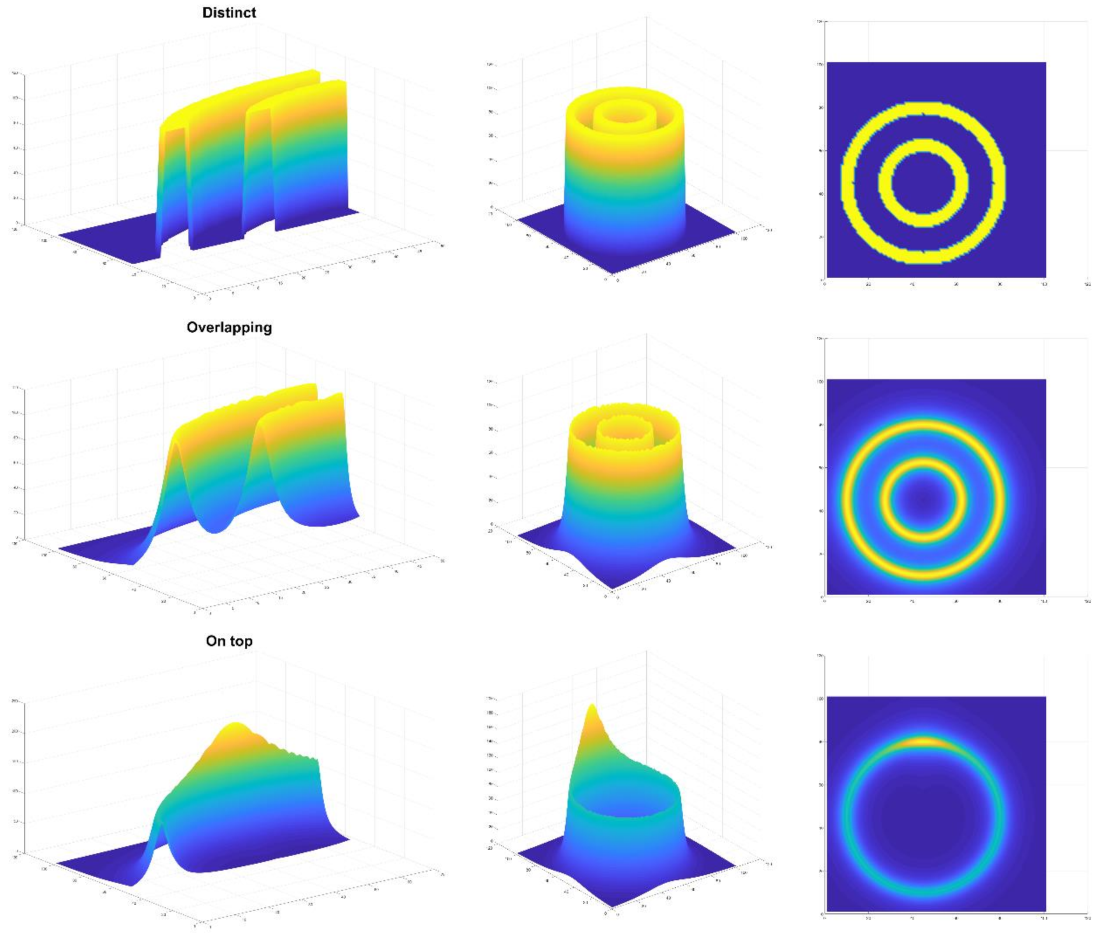

Figure (SI-2) 1. Three different model maps. For clarity equal intensity was used for the components.

Supplementary Information 3
Comparison of MCR-ALS and our method

'Triple top' model: In this model map, three different (slightly overlapping) spectra were mixed with different spatial distribution: ***spectrum-1*** (k_1= 1620 cm^{-1}, γ_1=60 cm^{-1}), ***spectrum-2*** (k_2=1850 cm^{-1}, γ_2=40 cm^{-1}), arranged as in the 'on top' model, and an additional ***spectrum-3*** (k_3=1450 cm^{-1}, γ_3=50 cm^{-1}). The 'on top' model was extended by adding ***spectrum-3*** in two additional 20-degree arcs at the east and west sides of the ***spectrum-1*** circle. The intensities of both ***spectrum-2*** and ***spectrum-3*** 'on top' spectra were 1% of that of the ***spectrum-1*** in the main circle. To the spectra, 1% white noise as compared to the maximum of ***spectrum-1*** was added. The analysis of the 'triple top' model is shown in Figure (SI-3) 1. Now, we had three significant maps (i.e. the rank of the data matrix is 3), so we used three V_i vectors and calculated 4 clusters. It is stunning how well the cluster spectra gave back the distribution of different component spectra. The cluster spectra are, however, very far from the pure component spectra. Each of them is dominated by the main ***spectrum-1*** (1620 cm^{-1}) and after careful examination, a tiny little shoulder can be observed at frequencies of the other two components (at 1450 cm^{-1}, and 1850 cm^{-1}) in the corresponding

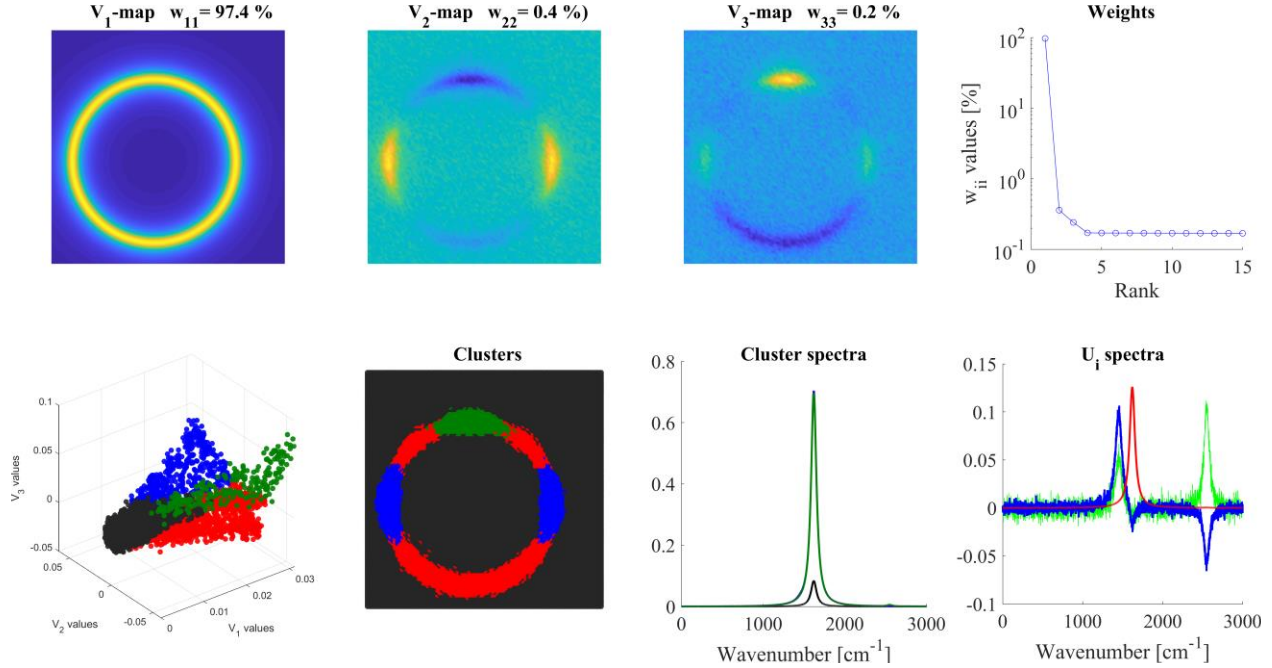

Figure (SI-3) 1. Triple on top model. For clustering 3 V_i maps were used and 4 clusters were calculated.

cluster spectra. U_i spectra, however, clearly indicate the presence and the frequencies of these minor components, but it is difficult to figure out how the U_i spectra should be combined in order to obtain separated component spectra.

To compare our method with other ones we have downloaded the MCR-ALS program from (https://mcrals.wordpress.com/download/mcr-als-2-0-toolbox/) and following the instructions, we applied MCR-ALS to this model map. The results are shown in Figure (SI-3) 2. MCR-ALS have found the main circle but none of the arcs was observable on the map given by the program output. We supplied the information that the program should look for 3 different components, but the spectra calculated by the program were not better than the SVD basis-spectra. The program has found the three frequencies of the components but the mixture was far from the real mixture that we have prepared for mapping. We have to

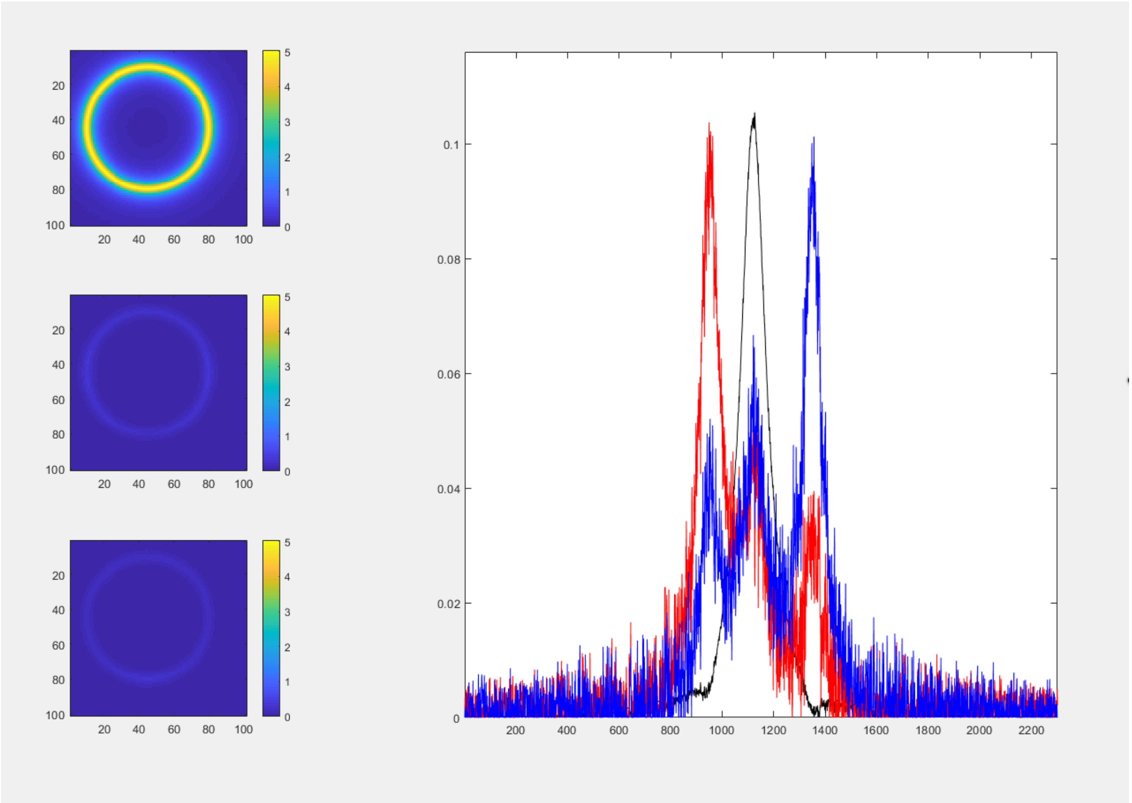

Figure (SI-3) 2. MCR-ALS calculation on 'triple on top' model map. Left panel: distribution of the component spectra on the map. Right panel: The three calculated component spectra.

admit, that we are not experienced users of MCR-ALS, maybe with some fine-tuning of the fitting parameters much better result would be possible.

Comparing MCR-ALS and our SVD based method we can conclude, that our new method is giving definite answers about the structural details of the map, which was not possible with MCR-ALS. Neither method was able to resolve the clear spectra of the components, although both methods gave correct information about the frequencies of the component spectra.

Supplementary information 4
Comparison of PCA-clustering and SVD-clustering

Earlier, a PCA clustering method was already published [3,4]. The two methods, although they seem to be very similar, differ considerably.

Although PCA 'scores', aside from a multiplication factor, give the same map as SVD V_i vectors and can be used for deducing information about the deviation from the average, there is a main difference in clustering. All the clustering methods are using some kind of distance between the points in the point group to be clusterized; therefore, the absolute value of coordinates of the data points are very important. The 'score' matrix in PCA can be calculated as the product of W and V matrices of SVD. Consequently, every V_i vector is multiplied with its singular value and since these singular values are different, every V_i vector is multiplied with a different number. The difference might be several orders of magnitudes. The clustering methods are using these 'scores' in PCA, and the V_i values in SVD for clustering, therefore, the clusters got from them will be completely different. A simple example is shown in Fig. (SI-4)-1.

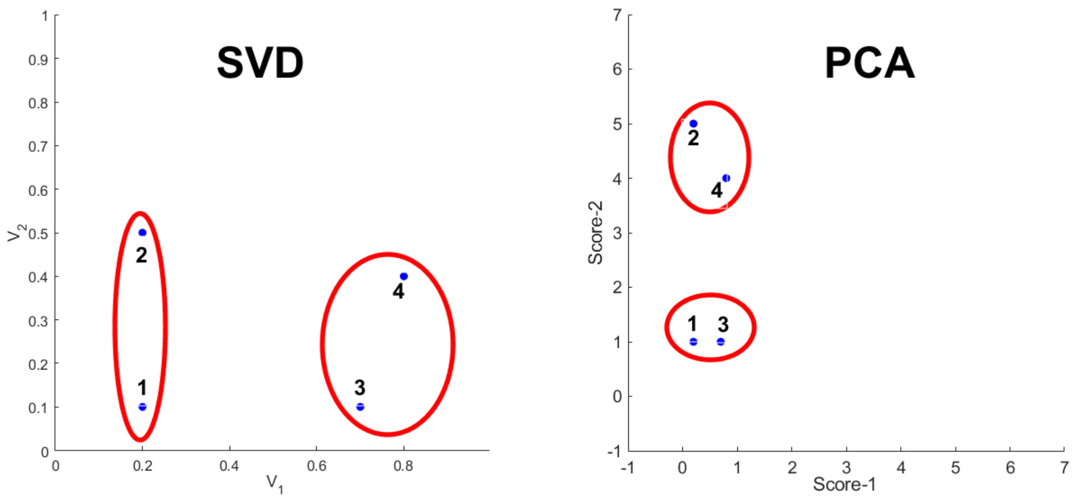

Fig. (SI-4)-1 Clustering of the same data set in SVD and in PCA. Note that the clusters contain different points in the two cases.

The question remains, which clustering is giving back better the structure of the sample. We applied both methods to our model maps (Fig. 1 and Fig. 3). In the case of the "distinct" model, there was no difference between the results of the two methods (Fig. (SI-4)-2 left panel). Both SVD and PCA-clustering behaved similarly. Both methods resolved the 1% structure (the inner circle) and at increasing noise, the disappearance of the inner circle was very similar.

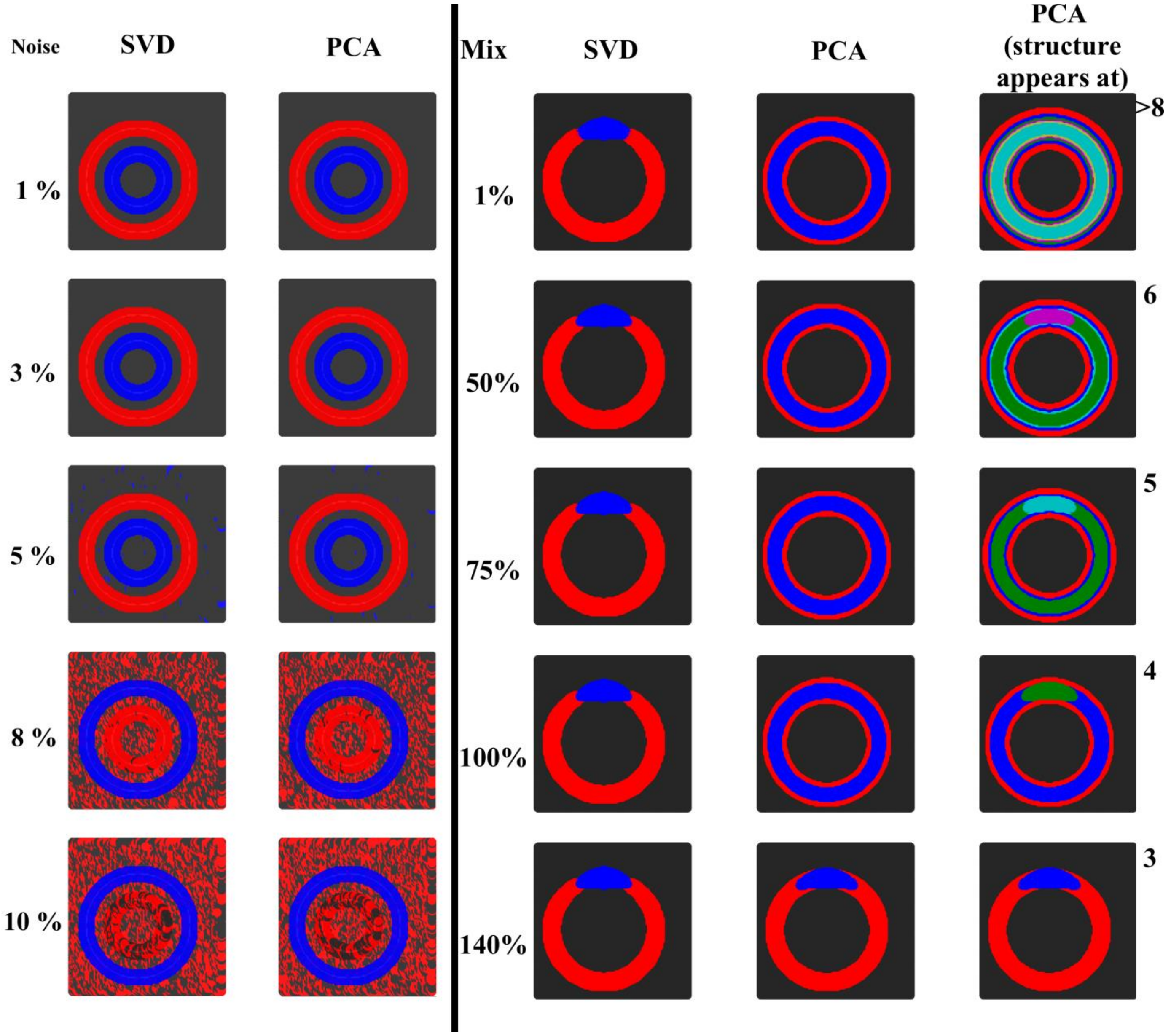

Fig. (SI-4)-2

Comparison of SVD and PCA clustering. The first two colums show the cluster maps for two distinctly arranged components (“distinct“ model) at increasing noise and assuming three clusters. The third and fourth columns show the cluster maps for two components on each other (“on top“ mondel) at increasing mixing intensity of the second component (on the top of the ring), also assuming three clusters. The fifth column shows, how many cluster had to be assumed at a given mixing ratio to make visible the “on top“ component.

The situation is completely different in the case of the “on top” model. In the case of PCA-clustering, the structure appears at three clusters only when the added (“on top”) component’s contribution to the amplitude at the maximum exceeds 140%. At lower contribution, the component disappears from the structure, clustering prefers the concentric circles. The last column of Fig. (SI-4)-2 shows that upon decreasing “on top“ contribution the re-appearance of the correct component location, requires the assumption higher and higher numbers of clusters (from bottom to top). At 1% noise even at 8 clusters

(the maximum number allowed in our software) the location of the added “on top“ component does not appear at all ((SI-4)-2).

As a conclusion, we can state, that SVD-clustering is giving back the structure – at least in model systems – much better for samples having spatial overlap between their different components. If the structures are distinct (no overlap between the components) the two methods perform similarly.

Thus, in real samples, the restriction of having only ‘distinct’ components, practically excludes the usability of the PCA-clustering.

If we, however, manipulate the PCA scores, they might behave similarly to SVD. Converting all PCA scores to zero mean and variance=1 is one of such manipulations. It is a linear transformation of the PCA scores (shifting of the scale and multiplication of the scale). SVD also can be regarded as such manipulation of PCA scores as well, since dividing the scores (multiplication of the scale) with the corresponding loadings (it is also a linear transformation) one will get the V_i vectors of SVD. The two methods in principle differ only in the numbers using for multiplication (since shifting does not affect the clustering), nevertheless the numbers have the same order of magnitude.

Supplementary information 5
Determination of the rank of SVD

We think that all methods defined so far suffer from subjectivism. In every method, there is a parameter that should be personally chosen. We have investigated what would be the result of different methods in one of our model systems where three components were put together. The no noise picture looks like this:

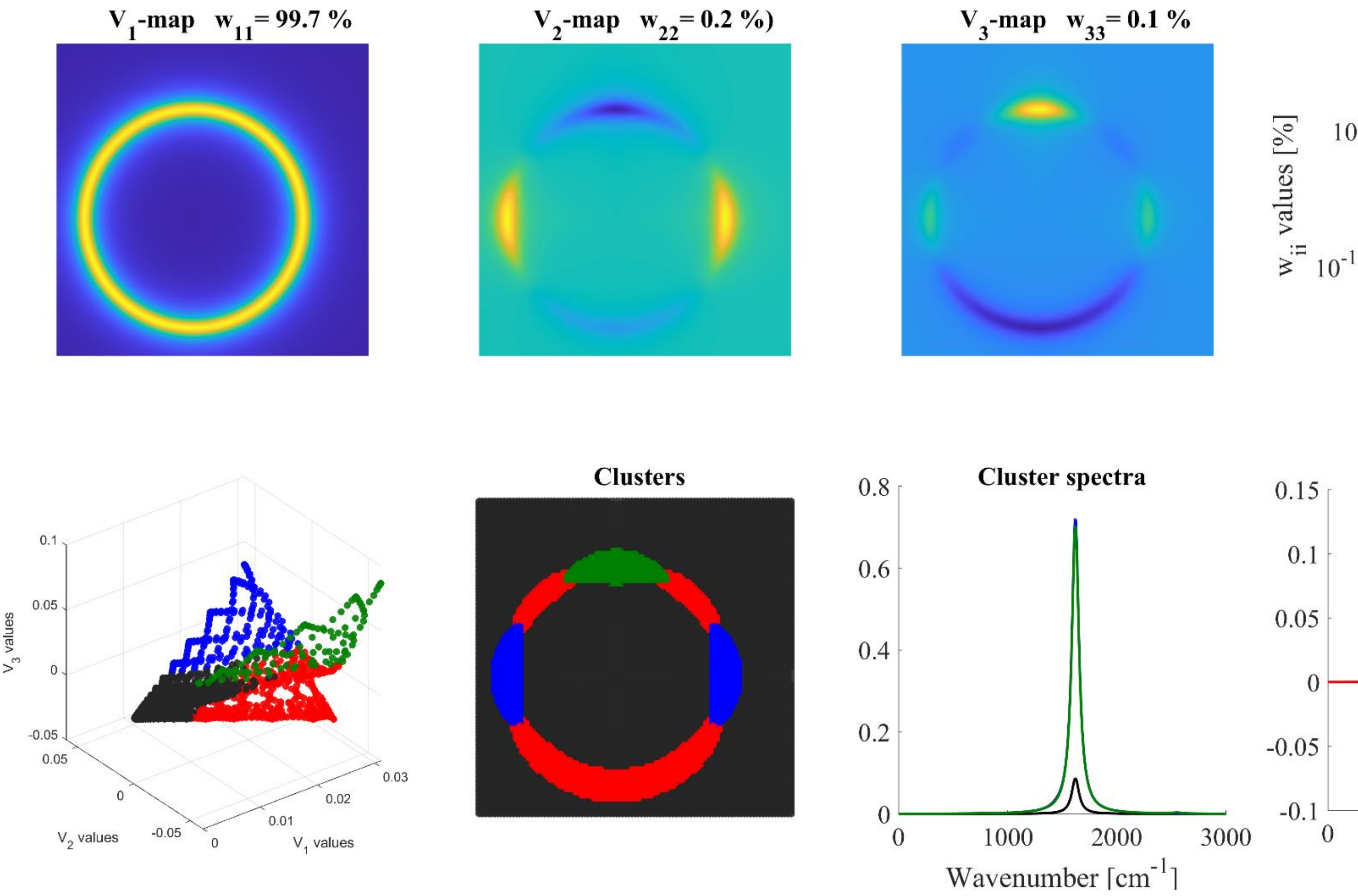

The two added components (at “north” and “west-east” sides of the ring) have a 0.5% amplitude of the full-ring component. All three components are very well resolved; the rank is 3 (W has three well-resolved components).

If we add 1% white noise the picture is slightly different:

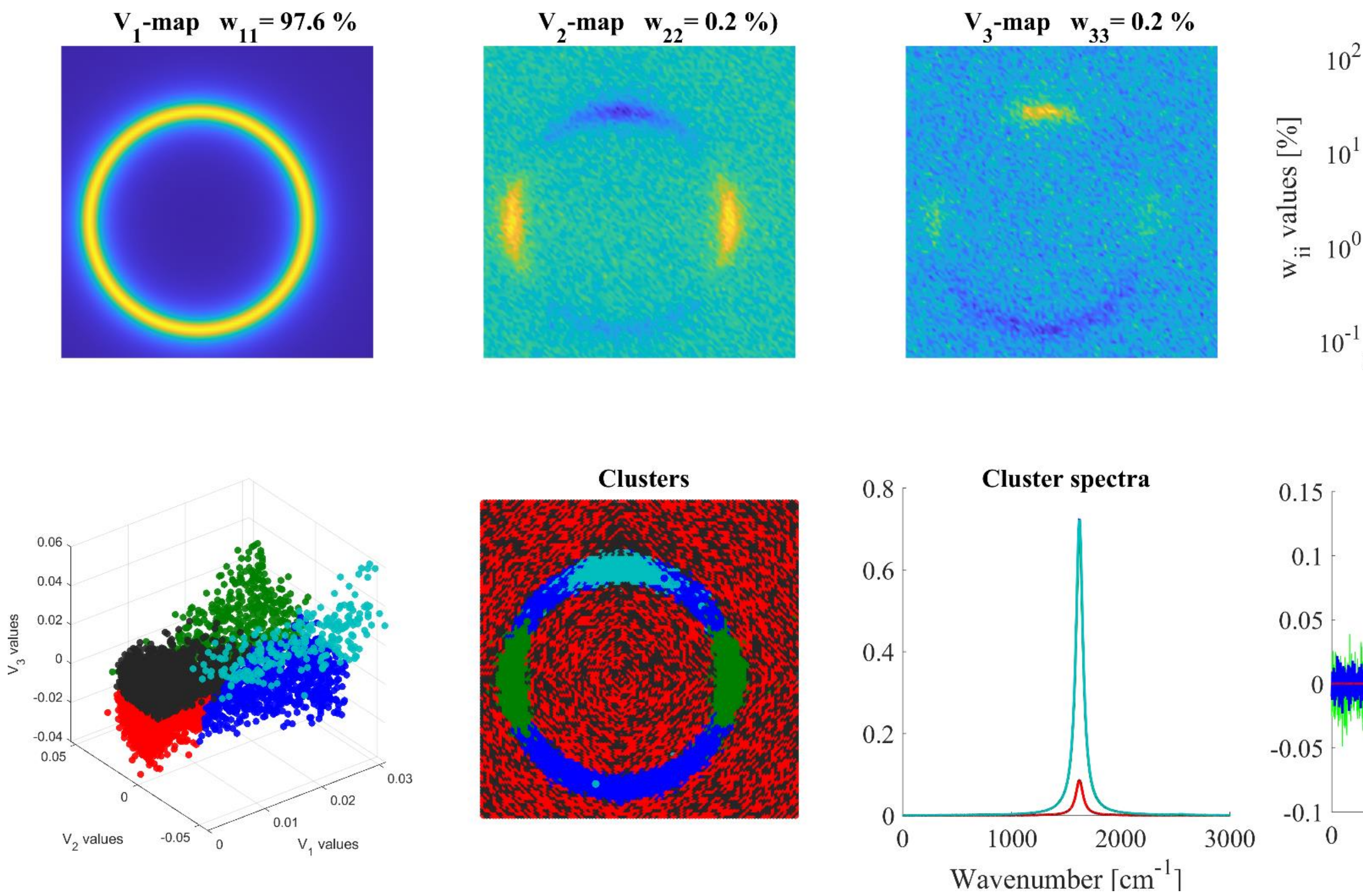

Looking just the W values one would say that the independent components are maximum two, but most likely one. Nevertheless, three V pictures show some structure.

Although there are several methods present in the literature to determine the rank of the data matrix we have tried only three different methods for both maps in order to estimate the correct rank of the data matrix.

a./ Malinowski method [5]

We have used the **sfa.m** Matlab file in order to calculate the rank of the data matrices. In this method, the arbitrary, subjective parameter is the significance level.

b./ Masia method [6]

The method according to the authors is: *„Assuming that at least half of the singular vectors are dominated by noise, we can determine this dependence by fitting a linear slope to the singular values with indices larger than $S/2$. We then classify singular values as being noise-dominated when they are less than a factor $sqrt(2)$ larger than the fit, i.e. when their root-mean square (RMS) signal is estimated to be less than the RMS noise, resulting in an index cutoff i_{max}."* [6].

Here the arbitrary (subjective) factor is the sqrt(2) times difference between the fitted and calculated singular values.

c./ Lobanova method (SVD-ADC)[7]

The method uses autocorrelation to determine the rank of the data matrix: "*for each left and right singular vectors, we calculate spectral R^S_i and spatial R^C_i autocorrelation coefficients at "one" pixel shift*" [7].

We have used the method with a 50% threshold (as it is given by the description in the paper, and it is an arbitrary number as well).

**Table I. Estimation of the rank of the data matrix using different methods**				
		**Estimated ranks**		**Reference**
**Method**	**Parameter choosen**	**0.5% effect, 0% noise**	**0.5% effect, 1% noise**	
Malinowskii method	Significance level (<5%)	17	1	5
Masia method	The alteration from the fit (>sqrt(2)	21	1	6
Lobanova method	Correlation factors of U and V (both >50%)	3	2	7

As a conclusion, according to our thorough investigation, no method (that has been described in the literature) is giving back the three sample components if noise is comparable or even higher than the smallest component. Our method (with an arbitrarily chosen rank), however, gives back the correct structure. We do not know how to determine exactly the rank, but seemingly we are not alone in the literature. We do not know better than a "trial and error" method as we tried to emphasize in the manuscript.

References

1 Szalontai, B. & Zimanyi, L. Chemometrics Meets Cytometry. Analysis of Multivariate Spectral Data to Organize and Discriminate Biological Information. *Cytom Part A* **85a**, 660-662, https://doi.org/10.1002/cyto.a.22493 (2014).

2 Henry, E. R. & Hofrichter, J. Singular Value Decomposition - Application to Analysis of Experimental-Data. *Methods in Enzymology* **210**, 129-192 (1992).

3 Koljenovic, S. *et al.* Tissue characterization using high wave number Raman spectroscopy. *J Biomed Opt* **10**, 031116, https://doi.org/10.1117/1.1922307 (2005).

4 Parthasarathy, R. *et al.* Application of multivariate spectral analyses in micro-Raman imaging to unveil structural/chemical features of the adhesive/dentin interface. *J Biomed Opt* **13**, 014020, https://doi.org/10.1117/1.2857402 (2008).

5 Malinowski, E. R. *Factor Analysis in Chemistry*. (Wiley, 2002).

6 Masia, F., Glen, A., Stephens, P., Borri, P. & Langbein, W. Quantitative Chemical Imaging and Unsupervised Analysis Using Hyperspectral Coherent Anti-Stokes Raman Scattering Microscopy. *Anal Chem* **85**, 10820-10828, https://doi.org/10.1021/ac402303g (2013).

7 Lobanova, E. G. & Lobanov, S. V. Efficient quantitative hyperspectral image unmixing method for large-scale Raman micro-spectroscopy data analysis. *Anal Chim Acta* **1050**, 32-43, https://doi.org/10.1016/j.aca.2018.11.018 (2019).
